# Dopaminergic stimulation leads B-cell infiltration into the central nervous system upon autoimmunity

**DOI:** 10.1186/s12974-021-02338-1

**Published:** 2021-12-17

**Authors:** Carolina Prado, Francisco Osorio-Barrios, Paulina Falcón, Alexandra Espinoza, Juan José Saez, María Isabel Yuseff, Rodrigo Pacheco

**Affiliations:** 1 Laboratorio de Neuroinmunología, Centro Ciencia & Vida, Fundación Ciencia & Vida, Avenida Zañartu #1482, Ñuñoa, 7780272 Santiago, Chile; 2 Facultad de Medicina y Ciencia, Universidad San Sebastián, Providencia, 7510156 Santiago, Chile; 3 Laboratory of Immune Cell Biology, Department of Cellular and Molecular Biology, Pontificia Universidad Católica de Chile, 8330025 Santiago, Chile

**Keywords:** Regulatory B lymphocytes, Antigen-presenting cells, Chemokine receptors, Neuroinflammation, Experimental autoimmune encephalomyelitis, Central nervous system homing

## Abstract

**Background:**

Recent evidence has shown dopamine as a major regulator of inflammation. Accordingly, dopaminergic regulation of immune cells plays an important role in the physiopathology of inflammatory disorders. Multiple sclerosis (MS) is an inflammatory disease involving a CD4^+^ T-cell-driven autoimmune response to central nervous system (CNS) derived antigens. Evidence from animal models has suggested that B cells play a fundamental role as antigen-presenting cells (APC) re-stimulating CD4^+^ T cells in the CNS as well as regulating T-cell response by mean of inflammatory or anti-inflammatory cytokines. Here, we addressed the role of the dopamine receptor D3 (DRD3), which displays the highest affinity for dopamine, in B cells in animal models of MS.

**Methods:**

Mice harbouring *Drd3*-deficient or *Drd3*-sufficient B cells were generated by bone marrow transplantation into recipient mice devoid of B cells. In these mice, we compared the development of experimental autoimmune encephalomyelitis (EAE) induced by immunization with a myelin oligodendrocyte glycoprotein (MOG)-derived peptide (pMOG), a model that leads to CNS-autoimmunity irrespective of the APC-function of B cells, or by immunization with full-length human MOG protein (huMOG), a model in which antigen-specific activated B cells display a fundamental APC-function in the CNS. APC-function was assessed in vitro by pulsing B cells with huMOG-coated beads and then co-culturing with MOG-specific T cells.

**Results:**

Our data show that the selective *Drd3* deficiency in B cells abolishes the disease development in the huMOG-induced EAE model. Mechanistic analysis indicates that although DRD3-signalling did not affect the APC-function of B cells, DRD3 favours the CNS-tropism in a subset of pro-inflammatory B cells in the huMOG-induced EAE model, an effect that was associated with higher CXCR3 expression. Conversely, the results show that the selective *Drd3 deficiency* in B cells exacerbates the disease severity in the pMOG-induced EAE model. Further analysis shows that DRD3-stimulation increased the expression of the CNS-homing molecule CD49d in a B-cell subset with anti-inflammatory features, thus attenuating EAE manifestation in the pMOG-induced EAE model.

**Conclusions:**

Our findings demonstrate that DRD3 in B cells exerts a dual role in CNS-autoimmunity, favouring CNS-tropism of pro-inflammatory B cells with APC-function and promoting CNS-homing of B cells with anti-inflammatory features. Thus, these results show DRD3-signalling in B cells as a critical regulator of CNS-autoimmunity.

**Supplementary Information:**

The online version contains supplementary material available at 10.1186/s12974-021-02338-1.

## Background

Dopamine has emerged as a major regulator of inflammation by stimulating dopamine receptors (DRs) in innate and adaptive immune cells [1, 2]. Remarkably, the stimulation of low-affinity DRs, including DRD1 and DRD2, by high levels of dopamine has been shown to exert anti-inflammatory effects in sepsis [3], Parkinson’s disease [4, 5], inflammatory bowel diseases [6, 7], and multiple sclerosis (MS) [8, 9]. Conversely, low dopamine levels, through the stimulation of high-affinity DRs, including DRD3, DRD4 and DRD5, have been shown to promote inflammation in animal models of Parkinson’s disease [10–13], inflammatory bowel diseases [14, 15], allergic asthma [16], and MS [17–20]. Interestingly, alterations in dopamine levels have been detected in the striatum in experimental autoimmune encephalomyelitis (EAE) [21–23]. Moreover, the dopaminergic system has been found to be altered in both innate and adaptive immune system in MS patients [19, 24, 25]. Since the reduction of striatal dopamine has been shown to induce an earlier onset of the disease and to increase EAE severity [22], it is likely that dopaminergic signalling through high-affinity DRs in immune cells infiltrating the brain might play a regulatory role in central nervous system (CNS) autoimmunity. According to this possibility, previous studies have addressed the role of DRD5 in dendritic cells (DCs) and CD4^+^ T cells. DRD5-signalling in DCs potentiated IL-12 and IL-23 production, thereby promoting Th1 and Th17-mediated autoimmunity in the CNS [17–19]. In addition, DRD5-signalling in CD4^+^ T cells exerted a dual role in the physiopathology of EAE, potentiating early inflammation mediated by effector T cells, but exacerbating suppressive activity in Treg cells and thereby dampening disease manifestation in late EAE stages [20]. Although dopaminergic signalling through DRD3, which displays the highest affinity for dopamine, has been strongly associated with the development and progression of inflammatory disorders [10, 12, 14, 15, 26], the involvement of this receptor in the physiopathology of MS/EAE remains poorly explored.

MS is a chronic inflammatory disorder involving an autoimmune response to the CNS characterized by demyelination, axonal degeneration, and gliosis [27, 28]. Several studies have consistently shown a major role of CD4^+^ T cells leading the damage of the CNS, whereas the involvement of B cells in the pathogenesis of MS was attributed for many years just to autoantibody production [29]. Nevertheless, current successful therapeutic approaches based on B-cell depletion have motivated investigations to improve our understanding of the role of B cells in the pathophysiology of MS [30–33]. Accordingly, the evidence has suggested that, in addition to the production of autoantibodies, B cells might play an essential role in triggering the autoimmune response as well as in the progression and recovery of the disease in animal models of MS [34–38]. Some studies performed in mice have indicated that B cells play an important role as antigen-presenting cells (APC) in the CNS, which is required for the re-stimulation of autoreactive T cells infiltrating the target tissue [37–40]. Moreover, another group of studies has shown that B cells participate as potent regulators of inflammation, providing both pro-inflammatory or immunosuppressive cytokines [34, 35, 38, 41, 42]. Notably, the production of regulatory cytokines by B cells might be induced by the recognition of autoantigens or by mechanisms independent of Ag-recognition.

The main animal model to study MS is the EAE, for which there are many variants. Two different EAE variants have been used to study different features of B cells in MS, which differ in the nature of the autoantigen [43, 44]. The first one is induced by immunization with a short peptide derived from the myelin oligodendrocyte glycoprotein (MOG_35-55_; pMOG). In this model, B cells may be activated independently of the B-cell receptor (BCR) specificity. Although in the pMOG-induced EAE model B cells are capable of presenting short peptides through direct binding to cell surface MHC-II molecules, their function is of secondary relevance as EAE develops with the same kinetic and intensity in wild-type mice and B-cell deficient mice [38]. Of note, the early depletion of B cells with an anti-CD20 antibody results in exacerbated disease manifestation, whilst the late treatment with anti-CD20 antibody reduces the disease severity in pMOG-induced EAE in C57BL/6 mice, indicating that B cells play an important regulatory role in this EAE model in this mouse strain [35]. Furthermore, these results indicate that despite APC-function of B cells is negligible in this EAE model, B cells still play a regulatory role, immunosuppressive at early-stage and pro-inflammatory at late-stage [35]. The second EAE model is induced by immunization with the full-length human MOG protein (huMOG), in which antigen-specific activated B cells play a primary role in the development of the disease, exerting a fundamental APCs function in the CNS, and also provide significant pro-inflammatory cytokines and participate as a source of antibody-producing plasma cells [38, 45–47].

Despite B cells play an important role in the physiopathology of MS [33] and constitute one of the leukocyte populations with the highest levels of DRs expression [48], the role of dopaminergic stimulation in B cells in CNS autoimmunity has not been yet studied. Indeed, B cells have been shown to express the highest levels of DRD3 [11, 48], whose stimulation has been strongly associated with inflammation [49]. Here we addressed the role of DRD3 in the adaptive immune system in CNS autoimmunity. Our findings indicate that DRD3-expression in CD4^+^ T cells was irrelevant in EAE, whilst DRD3 in B cells exerted fundamental regulatory processes in CNS autoimmunity. Mechanistic analysis revealed a dual role of DRD3 in B cells, promoting CNS-tropism of B cells with APC potential and favouring the infiltration of regulatory (suppressive) B cells into the CNS.

## Methods

### Animals

Six- to eight-week-old male or female mice of the C57BL/6 background were used in all experiments. Wild-type (WT, *Drd3*^+*/*+^), µMT (B-cell deficient), *Rag1*^*−/−*^ (B- and T-cell deficient) and 2D2 (bearing the transgenic TCR specific for the MOG_35-55_ peptide on IA^b^ molecules; TCR^MOG^) mice were purchased from The Jackson Laboratory (Bar Harbor, ME). DRD3-knockout (*Drd3*^*−/−*^) mice were kindly donated by Dr. Marc Caron [50] and B6.SJL.PTPRC (*Cd45.1*^+*/*+^, *Drd3*^+*/*+^) mice were kindly donated by Dr. Maria Rosa Bono [51]. Transgenic 2D2 *Drd3*^*−/−*^ mice were generated by crossing parental mouse strains.

### Generation of mixed-bone marrow chimera mice

Mice harbouring *Drd3* deficiency in specific cell populations were generated using the mixed-bone marrow (BM) chimera system, as described previously [34, 41]. Briefly, *Rag1*^*−/−*^ or µMT mice were irradiated with 1100 cGy γ-irradiation (Cs source), and 24 h later received the i.v. transfer of a mix of donor BM cells from indicated mouse strains (10^7^ total BM cells per recipient mouse). Female BM was used to transfer into male or female recipients, whilst male BM was used to transfer just into male recipients (to avoid incompatibility of Y-encoded genes by female recipients). Before EAE experiments, chimeric mice were left untreated for 8 weeks to let the hematopoietic system reconstitute the peripheral lymphoid system, as described before [20]. To restrict the genetic *Drd3 *deficiency to B and T cells, irradiated *Rag1*^*−/−*^ mice received a BM mix composed of 80% from *Rag1*^*−/−*^ mice and 20% from *Drd3*^*−/−*^ mice. The control group received a BM mix composed of 80% from *Rag1*^*−/−*^ mice and 20% from *Drd3*^+*/*+^ mice. Thus, all B and T cells were originated from the *Drd3*^*−/−*^ (or *Drd3*^+*/*+^ in the control group) BM. On the other hand, the 5:1 ratio (*Rag1*^*−/−*^-to-*Drd3*^*−/−*^ or *Rag1*^*−/−*^-to-*Drd3*^+*/*+^) in the donor BM [52] ensure that all the other hematopoietic lineages different from lymphocytes were predominantly originated from the *Rag1*^*−/−*^ BM, and therefore were *Drd3* sufficient. To restrict genetic *Drd3 *deficiency only to B cells, irradiated µMT mice received a BM mix composed of 80% from µMT mice and 20% from *Drd3*^*−/−*^ mice. The control group received a BM mix composed of 80% from µMT mice and 20% from *Drd3*^+*/*+^ mice. Thus, all B cells were originated from the *Drd3*^*−/−*^ (or *Drd3*^+*/*+^ in the control group) BM, whilst all the other hematopoietic lineages were predominantly originated from the µMT BM. FACS analysis confirmed hematopoietic reconstitution of sub-lethally irradiated mice. In some experiments, mixed BM chimera mice were generated by reconstituting irradiated µMT mice with a BM mix from B6.SJL.PTPRC *Drd3*^+*/*+^ (*Cd45.1*^*+/+*^) mice and BM from *Drd3*^*−/−*^ mice (*Cd45.2*^*+/+*^) at 50-to-50 or 30-to-70 ratios.

### EAE induction and evaluation

Experimental groups were generated by selecting mice at random while maintaining the same proportions of males and females and pairings by age. Mice displaying dwarfism and/or malformations were excluded. Experimental mice were s.c. immunized with 50 μg myelin oligodendrocyte glycoprotein 35–55 peptide (pMOG_35-55_; Genetel Laboratories, Madison, WI) or 100 μg human MOG protein (huMOG; Anaspec) emulsified in CFA (Invitrogen) supplemented with heat-inactivated *Mycobacterium tuberculosis* H37 RA (Difco Laboratories, Detroit, MI). In addition, mice received the i.p. administration of 500 ng pertussis toxin (Calbiochem, La Jolla, CA) on days 0 and 2. In primary-progressive EAE experiments, 24 h before disease induction *Rag1*^*−/−*^ mice received the i.v. transfer of 7,5 × 10^5^ splenic naïve (CD25^−^CD44^−^CD62L^+^) CD4^+^ T cells purified from *Drd3*^+*/*+^ or *Drd3*^*−/−*^ 2D2 transgenic mice. The clinical manifestation was assessed daily according to the following scoring criteria: 0, no detectable signs; 1, flaccid tail; 2, hind limb weakness or abnormal gait; 3, complete hind limb paralysis; 4, paralysis of fore and hind limbs; and 5, moribund or death.

The animals were included in the study when they underwent successful pMOG-CFA or huMOG-CFA immunization, defined by the formation of a sub-cutaneous emulsion at the site of injection. The animals were excluded: (i) when leakage of the emulsion was observed during injection; (ii) when infections or diseases unrelated to the experiment were detected, or when the animal died prematurely (usually less than 0.5% of the animals), avoiding the collection of disease severity data.

For the isolation of CNS mononuclear cells, mice were perfused through the left cardiac ventricle with cold PBS. The brain and spinal cord were dissected, and CNS tissue was minced into small pieces and digested by collagenase D (2.5 mg/ml; Roche Diagnostics) and DNase I (1 mg/ml; Sigma) at 37 °C for 45 min. Digested tissue was filtered through a 70 µm cell strainer obtaining single cell suspension that was subjected to centrifugation in a Percoll gradient (70%/30%). Mononuclear cells were removed from the interphase and resuspended in culture medium for further analysis. No blind protocol was carried out.

### Antibodies and flow cytometry analysis

For all analyses, live/dead discrimination was assessed using Zombie Aqua (ZAq) Fixable Viability kit (Biolegend). Spleens were minced until reach a cell suspension and then red blood cells were lysed using ammonium-chloride-potassium (ACK) buffer. Fluorochrome-conjugated mAb specific to mouse CD45 (clone 30-F11), CD19 (clone 6D5), IgD (clone 11-26c.2a), IgM (clone RRM-1), IL-6 (clone MP5-20F3), IL-10 (clone JES5-16E3), CXCR3 (clone CXCR3-A3), CD49d (clone R1-2), Tbet (clone 4B10), CD4 (clone GK-1.5), IFNγ (clone XMG1.2), IL-17 (clone TC11-18H10.1), GM-CSF (clone MP1-22E), CD20 (clone SA275A11), MHC-II (clone M5/114.15.2), CD21/CD35 (clone 7E9), CD1d (clone 1B1), CD5 (clone 53-73), CD23 (clone B3B4), CD138 (clone 281-2), CD44 (clone IM7), TCRβ (clone B183983), CD11c (clone N418), CD3 (clone 145-2C11), CD8 (clone 58-6,7), CD11b (clone M1/70), F4/80 (clone BM8), Ly6G (clone 1A8), CD45.1 (clone A20) and CD45.2 (clone 104) were purchased from Biolegend and to mouse Foxp3 (clone FJK-16 s) from eBioscience.

For the immunostaining of DRD2, DRD3, and DRD5, the rabbit anti-DRD2 antibody (ADR-002, Alomone labs), the rabbit anti-DRD3 antibody (ADR-003, Alomone labs), or the rabbit anti-DRD5 antibody (ADR-005, Alomone labs) was directly used or pre-incubated, respectively, with the antigenic peptide DRD2_11-26_ (DDLERQNWSRPFNGSE), with the antigenic peptide DRD3_15-29_ (CGAENSTGVNRARPH) or with the antigenic peptide DRD5_199-211_ (EEGWELEGRTENC) used to develop the antibodies (in a mixture of 0.8 mg/ml antibody and 0.4 mg/ml peptide) for 30 min as a control to abolish the specific immunostaining. For tyrosine hydroxylase (TH) immunostaining, the rabbit IgG anti-TH antibody (Santa Cruz Biotechnology; sc-14007) was used as primary antibody and compared with the rabbit irrelevant IgG (Santa Cruz Biotechnology; sc-3888) immunostaining as a negative control. Secondary goat anti-rabbit IgG-PE (50-8036) was obtained from TONBO Biosciences.

For intracellular immunostaining, cells were first labelled with antibodies specific for cell surface markers and then fixed and permeabilized with Foxp3 Fixation/Permeabilization kit (eBioscience). Next, Foxp3, Tbet, or TH immunostaining or intracellular cytokine immunostaining was performed in permeabilized cells followed by flow cytometry analysis. For analysis of cytokine production, cells were re-stimulated with ionomycin (Sigma) and PMA (Sigma) in the presence of brefeldin A (Invitrogen) for 5 h before immunostaining. All immunostainings were performed for 30 min at 4 °C. To quantify the absolute number of cells, 50 µL of 123 count eBeads (Thermo Fisher Scientific) was added to each sample prior to analysis by flow cytometry, and cell concentration was calculated using the following formula:

$$\mathrm{Cell concentration }\left(\mathrm{cells}/\mathrm{mL}\right)=\frac{\mathrm{Cell count}\times \mathrm{eBead volume}}{\mathrm{eBead count}\times \mathrm{cell volume}}\times \mathrm{eBead concentration}.$$

Data were collected with a FACSCanto II (BD) and results were analysed with FACSDiva (BD) and FlowJo software (Tree Star).

### In vitro B-cell culture

Splenic naïve B cells (TCRβ^−^CD11c^−^CD19^+^IgD^hi^IgM^int^) were isolated by cell-sorting from *Drd3*^+*/*+^ or *Drd3*^*−/−*^ mice. When proliferation was determined, naïve B cells were loaded with 5 µM Cell trace violet (CTV; Invitrogen, Carlsbad, CA, USA) before stimulation. Cells were stimulated with 10 µg/mL of goat anti-mouse IgM F(ab)’_2_ fragments (eBioscience); 1 µg/mL anti-CD40 (clone 1C10; Biolegend); 1 µM CpG DNA (ODN 1824; InvivoGen) and 10 ng/mL IFN-γ (Peprotech) for 5 d. Then, CXCR3, CD49d, Tbet expression, or cytokine production were determined by flow cytometry analysis. The extent of cell death was quantified using the ZAq Fixable Viability kit (Biolegend).

### In vitro antigen-presentation assays

huMOG protein or pMOG_35-55_ were coupled to glutaraldehyde-activated amino beads (40 × 10^6^ beads for 20 μg huMOG or pMOG) together with F(ab’)2 anti-mouse-IgM fragments in equal concentrations, as previously described [53]. Splenic CD19^+^ B cells were sorted from *Drd3*^+*/*+^ or *Drd3*^*−/−*^ animals and then incubated with huMOG- or pMOG_35-55_-coated beads in a B-cell-to-bead ratio 1:1 for 5 h at 37 °C to allow the uptake and processing of antigens. Next, B cells were washed and then incubated with MOG-specific CD4^+^ T cells at B-cell to T-cell ratio 1:5 for 5 days. For each point, 10^5^ CD4^+^ 2D2 T cells loaded with CTV were used. The proliferation and production of cytokines were evaluated by FACS analysis.

### Real-time quantitative PCR

Total RNA was extracted from cells using the Total-RNA EZNA kit (Omega Bio-Tek). The RNA was DNase-digested using the TURBO DNA-free kit (Ambion) and then used to synthesize cDNA catalysed by the M-MLV reverse transcriptase (Life Technologies). Quantitative gene-expression analysis was performed using Brilliant II SYBR Green QPCR Master Mix (Agilent). Expression of target genes was normalized to the levels of *gapdh* transcripts and multiplied by an arbitrary factor. *il10* Forward 5’-GAA GAC AAT AAC TGC ACC CA-3’; *il10* Reverse 5’-CAA CCC AAG TAA CCC TTA AAG TC-3’; *il6* Forward 5’-AGG ATA CCA CTC CCA ACA GAC CT-3’; *il6* Reverse 5’-CAA GTG CAT CGT TGT TCA TAC-3’; *csf2* Forward 5’-ACC ACC GCG GAT TTC AT-3’; *csf2* Reverse 5-TCA TTA CGC AGG CAC AAA AG-3’; *gapdh* Forward 5’-TCC GTG TTC CTA CCC CCA ATG-3’; *gapdh* Reverse 5’-GAG TGG GAG TTG CTG TTG AAG-3’.

### CD19 immunofluorescence analysis

Mice were perfused through the left cardiac ventricle with cold PBS, and then the brain was removed from the skull of each mouse. Then the brains were fixed with 4% paraformaldehyde for 48 h at 4 °C. Next, the tissues were dehydrated by immersion in 30% sucrose for 48 h at 4 °C. Then brains were embedded in optimal cutting temperature (OCT) compound and sagittally cut in 12 µm thick sections. Brain sections were mounted in glass-treated slides 76 × 26 mm (StarFrost©) and dried at 50 °C for 10 h. Then brain slices were hydrated with PBS, and epitope exposition was achieved by treatment with citrate buffer (pH 6.0) at 90 °C for 1 min. Afterwards, brain slices were washed three times with PBS for 5 min each, and then the slices were blocked and permeabilized with blocking solution (PBS containing 0.3% Triton and 5% BSA) for 1 h at room temperature. The brain slices were then incubated with anti-mouse CD19 conjugated to allophycocyanin (Thermo Fisher Scientific, CAT# 17-0193-82) diluted at 1:200 in blocking solution and co-incubated with Hoechst nuclear staining for 10 h at 4 °C. Next, the slices were washed three times with PBS in agitation for 10 min each. Finally, the brain slices were washed with distilled water and then mounted with Fluoromount mounting medium with glass coverslips. Images were acquired in an epifluorescence microscope at 10× and 20× objectives and analysed with the Fiji software.

### Statistical analyses and sample size estimation

The sample size was estimated with the mean and dispersion obtained from preliminary data using the sample size calculator: https://www.stat.ubc.ca/~rollin/stats/ssize/n2.html. A power of 80% was assumed. Using the same sample size calculator, post hoc analysis confirmed that all data have a power ≥ 75%. All values are expressed as the mean ± SEM. Statistical analysis was performed with two-tailed unpaired Student’s *t*-test when comparing only two groups and with one-way ANOVA followed by Tukey's post hoc test when comparing more than two groups with only one variable (treatment or genotype). To analyse differences in experiments comparing different genotypes and different treatments, two-way ANOVA followed by Sidak's post hoc test was performed. All analyses were carried out using the GraphPad Prism 6 Software. P values < 0.05 were considered significant.

## Results

### DRD3-stimulation in the adaptive immune system is required to the development of CNS autoimmunity

**Fig. 1:**
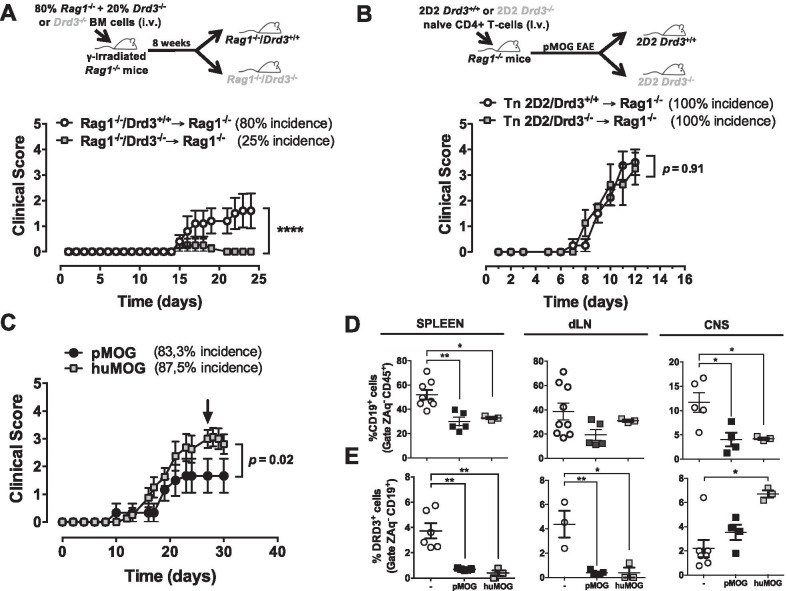
DRD3-signalling in lymphocytes is required for the development of CNS autoimmunity. **A** BM chimeric mice harbouring *Drd3*-deficient or *Drd3*-sufficient lymphocytes were generated by the i.v. transfer of a 4:1 mixed BM from *Rag1*^*−/−*^ and *Drd3*^*−/−*^ mice (grey symbols) or 4:1 mixed BM from *Rag1*^*−/−*^ and *Drd3*^+*/*+^ mice (white symbols), respectively, into γ-irradiated *Rag1*^*−/−*^ recipient mice. Afterwards, EAE was induced in chimeric mice by immunization with pMOG_35-55_ in CFA followed by pertussis toxin injection and disease severity was determined throughout the time-course of the disease development. *n* = 4–5 mice per group. Top panel shows an illustration of the experimental strategy for generation of chimeric mice. Bottom panel shows the quantification of clinical score for different experimental groups. (**B**) Primary progressive EAE was induced in mice bearing *Drd3*-deficient (grey symbols) or *Drd3*-sufficient (white symbols) CD4^+^ T cells by the i.v. transfer of transgenic naïve CD4^+^ T cells (Tn; 7.5 × 10^5^ cells per mouse) isolated from *Drd3*^*−/−*^ 2D2 or *Drd3*^+*/*+^ 2D2 mice into *Rag1*^*−/−*^ recipient mice. Disease severity was determined throughout the time-course of the disease development. *n* = 4 mice per group. Top panel illustrates the experimental design to induce primary-progressive EAE. Bottom panel shows the quantification of clinical score for different experimental groups. **C** EAE was induced in wild-type C57BL/6 mice by immunization with pMOG_35-55_ (black symbols) or huMOG (grey symbols) in CFA followed by pertussis toxin injection. Disease severity was evaluated throughout the time-course of the disease development. *n* = 6–8 mice per group. **D**, **E** At the peak of disease severity (indicated by an arrow in **C**), mononuclear cells were isolated from the spleen, draining lymph nodes (dLN) and central nervous system (CNS) and the frequency of CD19^+^ B cells from the CD45^+^ gate (**D**) and the percentage of DRD3 expression in CD19^+^ B cells (**E**) were evaluated. A control group (white symbols) without immunization (-) was included in the analysis. **D**, **E** *n* = 3–9 mice per group. **A**–**E** Each symbol represents data obtained from an individual mouse. The mean ± SEM are depicted. *, *p* < 0.05; **, *p* < 0.01; ****, *p* < 0.0001 by Mann–Whitney *U* test (**A**–**C**) or one-way ANOVA followed by Tukey’s post hoc *t*-test (**D**, **E**)

To address the role of DRD3 in the adaptive immune system in the development of CNS autoimmunity we generated chimeric mice bearing *Drd3*-deficient adaptive immune system and *Drd3*-sufficient innate immune system. For this purpose, recombination-activating gene 1 knockout (*Rag1*^*−/−*^) mice, which are devoid of T and B lymphocytes, were γ-irradiated with a myeloablative dose and then received the transfer of donor bone marrow (BM) obtained from *Rag1*^*−/−*^ mice and *Drd3*^*−/−*^ mice mixed in a 5:1 ratio *Rag1*^*−/−*^-to-*Drd3*^*−/−*^ as described before [34]. Thus, after BM reconstitution, *Drd3 deficiency* was confined to T and B lymphocytes, whilst non-lymphocytic hematopoietic cells were mostly originated from *Drd3*-sufficient (*Rag1*^*−/−*^) BM. The control group of mice, harbouring *Drd3*-sufficient adaptive immune system, received the transfer of BM obtained from *Rag1*^*−/−*^ mice (80%) and *Drd3*^+/+^ mice (20%). Eight weeks after BM-transfer, EAE was induced, and the extent of disease severity was evaluated throughout the time-course of disease development. The results show that EAE manifestation was abrogated in mice bearing *Drd3*-deficient adaptive immune system (Fig. 1A), indicating that DRD3 in T and/or B lymphocytes was required for EAE development. No differences were observed between males and females from the same experimental groups. Of note, DRD3-signalling in CD4^+^ T cells has been shown to promote inflammation in animal models of Parkinson’s disease [10–12] and inflammatory bowel diseases [14, 15]. Accordingly, we next evaluated the role of DRD3 in autoreactive CD4^+^ T cells in a mouse model of primary-progressive MS. To this end, 24 h before EAE induction, *Rag1*^*−/−*^ mice received the transfer of naïve CD4^+^ T cells isolated from *Drd3*-deficient or *Drd3*-sufficient 2D2 transgenic mice, which express a transgenic TCR specific for the MOG_35-55_ peptide (pMOG) on IA^b^ molecules [54]. The results show that mice receiving *Drd3*-deficient or *Drd3*-sufficient CD4^+^ T cells developed EAE with similar kinetics and severity (Fig. 1B), thus ruling out a relevant role of DRD3 in CD4^+^ T cells in the development of CNS autoimmunity. No differences were observed between males and females from the same experimental groups. We next attempted to determine the role of DRD3 in B cells, which have been associated with the production of pro-inflammatory and anti-inflammatory cytokines in EAE [35] and have been involved as key APC in the development of CNS autoimmunity [38]. Of note, our immunofluorescence analysis of CD19^+^ cells in brain slices shows that the extent of B-cell infiltration into the CNS significantly correlates with the clinical EAE score (Additional file 1: Fig. S1). Accordingly, to analyse the protective and pathogenic potential DRD3-signalling in B cells in CNS autoimmunity, we used two EAE models, which differ in the nature of the autoantigen and display differential B-cell involvement: (i) the EAE model independent of the APC-function of B cells, which is induced by immunization with pMOG; and (ii) the EAE model dependent of the APC-function of B cells, which is induced by immunization with huMOG. In this regard, we first compared the time-course of clinical manifestation, and the extent of DRD3 expression and B-cell frequency in relevant tissues when EAE was induced by immunization of WT mice with pMOG or huMOG. The results show that EAE manifestation was similar in both models, although the peak of severity was higher in huMOG-induced EAE than pMOG-induced EAE (Fig. 1C). No differences were observed between males and females from the same experimental groups. In addition, in both models, B-cell frequency was significantly reduced in the spleen and the CNS, whereas it remained unchanged in the cervical lymph nodes (CNS draining lymph nodes; dLN) during the peak of disease manifestation (Fig. 1D). Interestingly, the frequency of DRD3-expression was selectively increased in B cells infiltrating the CNS upon huMOG-induced EAE but not upon pMOG-induced EAE (Fig. 1E). Conversely, the percentage of DRD3 expression was reduced to a similar extent in dLN and splenic B cells in both EAE models (Fig. 1E; see the gating strategy for the analysis of DRD3 expression in Additional file 1: Fig. S2A). Interestingly, the density of DRD3 expression in B cells infiltrating the CNS was selectively reduced upon pMOG-induced but not in huMOG-induced EAE (Additional file 1: Fig. S2B, C). Nonetheless, the density of DRD3 expression was reduced in those B cells circulating through peripheral blood and the spleen upon both pMOG-induced or huMOG-induced EAE (Additional file 1: Fig. S2B, C). Further analysis of DRD3-expression in different B-cell subsets indicated that DRD3 upregulation observed in B cells infiltrating the CNS upon huMOG-EAE occurred selectively in CD20^+^ MHC-II^+^ B cells (Additional file 1: Fig. S3), which display APC-function [38]. In addition to CD20^+^ MHC-II^+^ B cells, we found that CD21^+^ CD23^+^ IgM^+^ B cells, which have been described to exert suppressive function [55], was the only B-cell subset expressing detectable levels of DRD3 upon pMOG-induced EAE in the CNS and dLN (Additional file 1: Fig. S3B-D). Taken together, these results suggest that DRD3 in B cells plays an important role in the development of CNS autoimmunity, involving different B-cell subsets depending on the nature of the antigen.

### *Drd3* deficiency in B cells exacerbates disease severity in an EAE model that does not depend on the APC-function of B cells

**Fig. 2:**
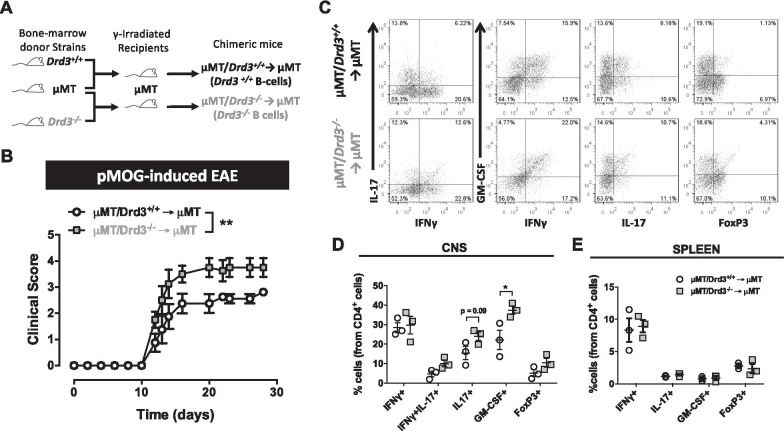
DRD3-signalling in B cells attenuates disease severity in an EAE model that does not depend on the APC-function of B cells. BM chimeric mice harbouring *Drd3*-deficient or *Drd3*-sufficient B cells were generated by the i.v. transfer of a 4:1 mixed BM from μMT and *Drd3*^*−/−*^ mice (grey symbols) or 4:1 mixed BM from μMT and *Drd3*^+*/*+^ mice (black symbols), respectively, into γ-irradiated μMT recipient mice. **A** Schematic illustration of chimeric mice generation. **B**–**E** EAE was induced in chimeric mice by immunization with pMOG_35-55_ in CFA followed by pertussis toxin injection. **B** Disease severity was evaluated throughout the time-course of the disease development. Values represent the mean ± SEM; *n* = 8 mice per group. **C**–**E** At the peak of disease severity (day 15 post-induction), mononuclear cells were isolated from the CNS (**C**, **D**) and the spleen (**E**) followed by ex vivo stimulation with PMA/ionomycin in the presence of brefeldin A, and intracellular cytokine staining analysis in CD4^+^ T cells was carried out by flow cytometry. **C** Representative dot-plots in the CD4^+^ gate are shown. Numbers indicate the percentage of cells in the corresponding quadrant. **D**, **E** Quantification of the frequency of CD4^+^ T cells producing IFNγ, IL-17, GM-CSF or expressing FoxP3; *n* = 3 mice per group. Each symbol represents data obtained from an individual mouse. The mean ± SEM are depicted. Data representative from one out of two independent experiments are shown. *, *p* < 0.05; **, *p* < 0.01; by Mann–Whitney *U* test (**B**) or two-way ANOVA followed by Sidak’s post hoc test (**D**, **E**)

To confirm the relevance of DRD3 in B cells in CNS autoimmunity, we next generated mice harbouring *Drd3* deficiency restricted only to B cells. For this purpose, µMT mice, which are devoid of B cells [56], were γ-irradiated with a myeloablative dose and then received the i.v. transfer of mixed BM progenitors coming from µMT (80%) and *Drd3*^*−/−*^ or *Drd3*^+*/*+^ (20%) mice (Fig. 2A). Since B cells arose from *Drd3*^*−/−*^ BM progenitors and all the other cells arose predominantly from µMT BM progenitors in mice bearing µMT/*Drd3*^*−/−*^ BM (µMT/*Drd3*^*−/−*^ → µMT mice) after reconstitution of the haematopoietic system, *Drd3* deficiency was restricted to B cells in these mice. Conversely, all haematopoietic system was *Drd3*^+*/*+^ in control mice bearing µMT/*Drd3*^*−*+*/*+^ BM (µMT/*Drd3*^+*/*+^ → µMT mice) (Fig. 2A). Of note, the frequencies of the main leukocyte populations were equivalent in BM chimera mice containing either *Drd3*^+/+^ or *Drd3*^−/−^ B cells (Additional file 1: Fig. S4). Using these chimeric mice, we first attempted to determine the relevance of DRD3 in B cells in CNS autoimmunity in a model that does not depend on the APC-function of B cells. Accordingly, EAE was induced with pMOG in µMT/*Drd3*^*−/−*^ → µMT and µMT/*Drd3*^+*/*+^ → µMT mice, and the disease severity as well as the phenotype of CD4^+^ T cells infiltrating the CNS were determined at the peak of disease manifestation. The results show that EAE severity was significantly higher in µMT/*Drd3*^*−/−*^ → µMT mice when compared with control µMT/*Drd3*^+*/*+^ → µMT mice (Fig. 2B). No differences were observed between males and females from the same experimental groups. According to the exacerbated disease manifestation, µMT/*Drd3*^*−/−*^ → µMT mice displayed an increased frequency of CD4^+^ Tcells producing GM-CSF in the CNS (Fig. 2C, D), which has been described to be the most inflammatory cytokine promoting neuroinflammation in EAE [57]. Conversely, no significant alterations were detected in the phenotype of peripheral CD4^+^ T cells when comparing both experimental groups of mice (Fig. 2E). Interestingly, it has been previously described that B cells express not only the DRD3, but also the DRD2 and DRD5 [48]. Moreover, previous works have shown that some immune cells express the machinery to synthesize dopamine [1, 17, 58], thus raising the possibility that this neurotransmitter might be released as an immunomodulator. To address the possibility that the effect observed in EAE in those mice harbouring *Drd3*-deficient B cells is due to altered expression of other DRs or to changes in the ability to synthesize dopamine, we compared the expression levels of DRD2, DRD5, and tyrosine hydroxylase (TH) in *Drd3*-sufficient and *Drd3*-deficient B cells. The results show a similar extent of DRD2 and DRD5 expression in both genotypes (Additional file 1: Fig. S5), thus ruling out the possibility that the effect observed on EAE development in those mice bearing *Drd3*-deficient B cells is due to altered expression of other DRs. Furthermore, our analysis shows that TH expression was barely detectable in B cells with both genotypes (Additional file 1: Fig. S5), suggesting that dopamine-derived from B cells does not play a relevant role in the effect observed in EAE in those mice harbouring *Drd3*-deficient B cells. Thus, these results together indicate that DRD3-signalling in B cells limits the generation of CD4^+^ T cells producing GM-CSF in the CNS and consequently attenuates disease manifestation in a model independent of the APC-function of B cells.

### DRD3 in B cell is required for disease manifestation in an EAE model that depends on the APC-function of B cells

**Fig. 3:**
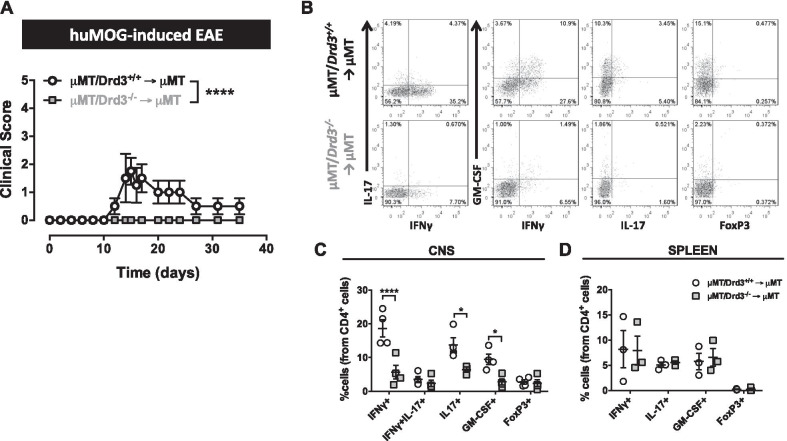
*Drd3* deficiency in B cell abrogates disease manifestation in an EAE model that depends on the APC-function of B cells. BM chimeric mice harbouring *Drd3*-deficient (grey symbols) or *Drd3*-sufficient (black symbols) B cells were generated as described in Fig. 2A. Next, EAE was induced in chimeric mice by immunization with huMOG in CFA followed by pertussis toxin injection. **A** Disease severity was evaluated throughout the time-course of the disease development. Values represent the mean ± SEM; *n* = 4 mice per group. **B**–**D** At the peak of disease severity (day 15 post-induction), mononuclear cells were isolated from the CNS (**B** and **C**) and the spleen (**D**) followed by ex vivo stimulation with PMA/ionomycin in the presence of brefeldin A, and intracellular cytokine staining analysis in CD4^+^ Tcells was carried out by flow cytometry. **B** Representative dot-plots in the CD4^+^ gate are shown. Numbers indicate the percentage of cells in the corresponding quadrant. **C**, **D** Quantification of the frequency of CD4^+^ T cells producing IFNγ, IL-17, GM-CSF or expressing FoxP3. *n* = 3–4 mice per group. Each symbol represents data obtained from an individual mouse. The mean ± SEM are depicted. Data representative from one out of two independent experiments are shown. *, *p* < 0.05; **, *p* < 0.01; ****, *p* < 0.0001 by Mann–Whitney *U* test (**A**) or two-way ANOVA followed by Sidak’s post hoc test (**C**, **D**)

We next evaluated the role of DRD3 in B cells in a model of CNS autoimmunity that depends on the APC-function of B cells. To this end, EAE was induced in µMT/*Drd3*^*−/−*^ → µMT and µMT/*Drd3*^+*/*+^ → µMT mice (Fig. 2A) using huMOG and disease severity as well as the extent of T-cell infiltration into the CNS were analysed. Strikingly, *Drd3 deficiency* in B cells abolished EAE manifestation (Fig. 3A). No differences were observed between males and females from the same experimental groups. According to the absence of disease manifestation in mice harbouring *Drd3*-deficient B cells, we observed that *Drd3* deficiency resulted in a sharp reduction of the frequency of inflammatory CD4^+^ Tcells in the CNS, including Th1, Th17, and GM-CSF producers CD4^+^ T cells (Fig. 3B, C). Nevertheless, the frequency of different CD4^+^ T-cell subsets in the periphery was not altered by *Drd3* deficiency in B cells (Fig. 3D). Taken together these results provide evidence that DRD3 in B cells plays a pro-inflammatory role in a model of CNS autoimmunity that depends on the APC-function of B cells, favouring the participation of inflammatory CD4^+^ T cells in the CNS and, thereby, promoting disease development.

### DRD3-signalling favours the expression of α4-integrin and increases the infiltration of immunosuppressive B cells into the CNS in an EAE model that does not depend on the APC-function of B cells

**Fig. 4:**
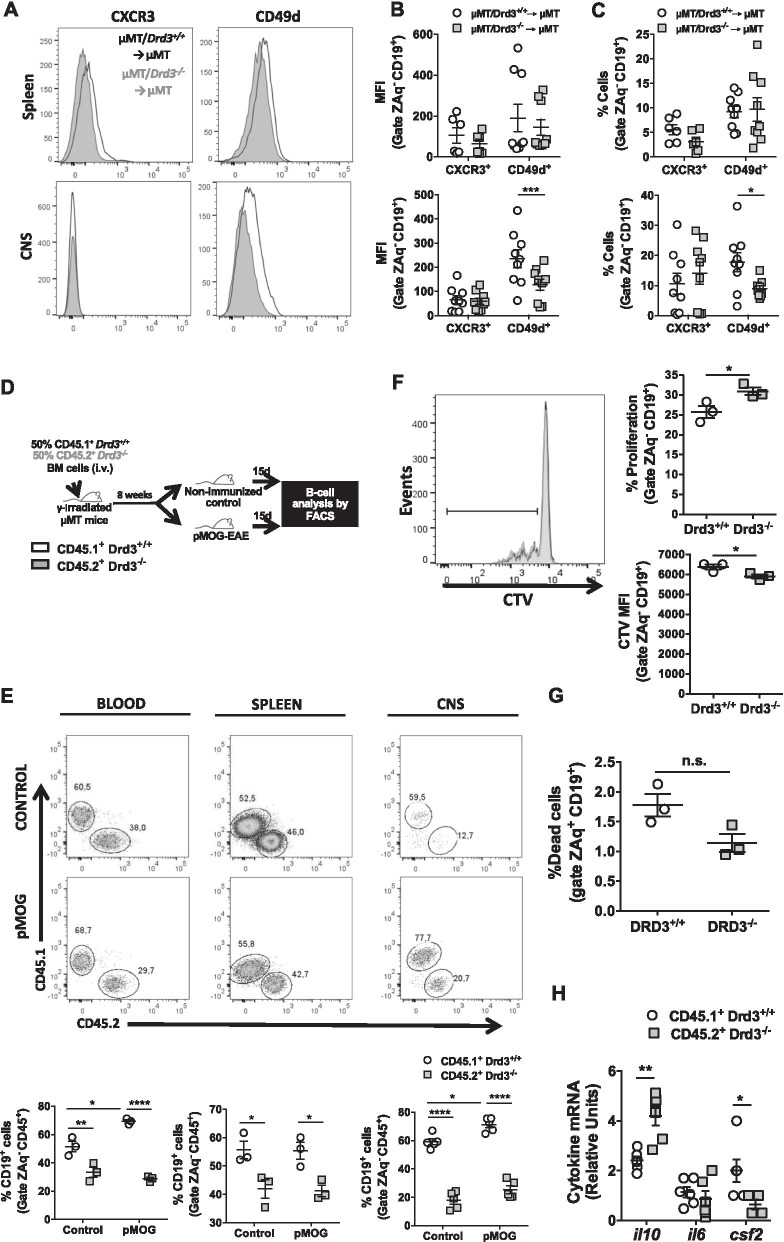
DRD3-signalling favours the expression of α4-integrin and attenuates the immunosuppressive profile in B cells infiltrating the CNS in an EAE model that does not depend on the APC-function of B cells. **A**–**C** BM chimeric mice harbouring *Drd3*-deficient (grey symbols) or *Drd3*-sufficient (white symbols) B cells were generated as described in Fig. 2A. Afterwards, EAE was induced in chimeric mice by immunization with pMOG_35-55_ in CFA followed by pertussis toxin injection. *n* = 6–9 mice per group. At the peak of disease severity (day 15 post-induction), mononuclear cells were isolated from the spleen and the CNS and the surface expression of CXCR3 and α4 integrin (CD49d) were analysed in the CD19^+^ population by flow cytometry. **A** Representative histograms for the expression of CXCR3 and CD49d in the CD19^+^ cells are shown. **B** Quantification of the mean fluorescence intensity (MFI) associated to the surface expression of CXCR3 and CD49d in living (ZAq^−^) CD19^+^ cells isolated from the spleen (top panel) and CNS (bottom panel). **C** Quantification of the percentage of surface expression of CXCR3 and CD49d in living (ZAq^−^) CD19^+^ cells isolated from the spleen (top panel) and CNS (bottom panel). (B-C) Each symbol represents data obtained from an individual mouse. The mean ± SEM are depicted. *, *p* < 0.05; ***, *p* < 0.001 by two-way ANOVA followed by Sidak’s post hoc test. (D-E) BM chimeric mice harbouring *Drd3*-deficient and *Drd3*-sufficient B cells were generated by the i.v. transfer of a 1:1 mixed BM from *Cd45.1*^+*/*+^*/Cd45.2*^*−/−*^*/Drd3*^+*/*+^ mice (white symbols) and *Cd45.1*^*−/−*^*/Cd45.2*^+*/*+^*//Drd3*^*−/−*^ mice (grey symbols) into γ-irradiated μMT recipient mice. Next, EAE was induced in chimeric mice by immunization with pMOG_35-55_ in CFA followed by pertussis toxin injection. **D** Schematic illustration of the experimental design. **E** At the peak of disease severity (day 15 post-induction), mononuclear cells were isolated from peripheral blood (left panel), the spleen (middle panel) and the CNS (right panel) and the frequency of total CD19^+^ B cells was analysed by flow cytometry. Top panels show representative dot-plots of CD45.1^+^ versus CD45.2^+^ cells in the CD19^+^ gate. Numbers indicate the percentage of cells in the corresponding region. Bottom panels show the percentage quantification. *n* = 3–5 mice per group. Each symbol represents data obtained from an individual mouse. The mean ± SEM are depicted. Data representative from one out of two independent experiments are shown. *. P < 0.05; **, *p* < 0.01, ****, *p* < 0.0001 by two-way ANOVA followed by Sidak’s *post hoc* test. **F**, **G** Naïve B cells (CD19^+^ IgD^hi^ IgM^int^ CD11c^−^ TCRβ^−^) were isolated from the spleen of *Drd3*-deficient (grey symbols/histograms) or *Drd3*-sufficient (white symbols/histograms) mice by cell-sorting, loaded with cell-trace violet (CTV) and incubated in vitro in the presence of anti-CD40, anti-IgM, IFNγ and the TLR9-ligand CpG for 5 days. **F** The extent of proliferation was evaluated as the dilution of the fluorescence associated to CTV in living (ZAq^−^) CD19^+^ cells by flow cytometry. Representative histograms are shown in the left panel. The marker indicates cells displaying dilution of CTV-associated fluorescence. Quantification of the percentage of cells displaying diluted CTV-associated fluorescence (top right panel) and the MFI of CTV-associated fluorescence (bottom right panel) are shown. **G** The extent of cell dead was determined as the percentage of ZAq^+^ cells in the CD19^+^ gate. **F**, **G** Each symbol represents data obtained from an individual mouse; *n* = 3 mice per group. The mean ± SEM are depicted. *, *p* < 0.05 by unpaired two-tailed Student’s *t*-test. n.s. non-significant. (**H**) Chimeric mice were treated as shown in **D** and at the peak of disease severity (day 15 post-induction), CD19^+^ B cells were isolated from the spleen and the levels of cytokine transcripts was analysed by qRT-PCR. The levels of *gapdh* transcripts were used as a housekeeping. Data were obtained from 4–6 mice per group. Each symbol represents data obtained from an individual mouse. The mean ± SEM are depicted. Data representative from one out of two independent experiments are shown. *, *p* < 0.05; **, *p* < 0.01 by two-way ANOVA followed by Sidak’s post hoc test

Next, we attempted to determine the mechanism by which DRD3 in B cells exerts a suppressive effect on the development of CNS autoimmunity in a model independent of the APC-function of B cells. Since *Drd3* deficiency in B cells affected the T-cell response only in the CNS, but not in the spleen (Fig. 2D, E), we addressed the possibility that DRD3-signalling was affecting B-cell migration into the CNS. In this regard, both α4-integrin (CD49d) and the chemokine receptor CXCR3 [59] have been involved in the infiltration of B cells into the CNS upon autoimmunity. Accordingly, EAE was induced with pMOG in µMT/*Drd3*^*−/−*^ → µMT and µMT/*Drd3*^+*/*+^ → µMT mice, and the extent of α4-integrin and CXCR3 expression was quantified in B cells infiltrating the CNS and the spleen (see the gating strategy in Figure S6). Whereas similar levels of α4-integrin and CXCR3 expression was detected in splenic B cells, a significant and selective reduction in the frequency and density of α4-integrin expression was observed in B cells infiltrating the CNS from µMT/*Drd3*^*−/−*^ → µMT mice when compared with µMT/*Drd3*^+*/*+^ → µMT mice (Fig. 4A–C). To confirm whether DRD3-signalling actually favours the selective α4-integrin expression on B cells, we performed in vitro experiments in which *Drd3*^+*/*+^ or *Drd3*^*−/−*^ B cells were activated in the presence or the absence of dopamine, and the extent of CXCR3 and α4-integrin expression was analysed. The results show that B-cell stimulation with dopamine enhanced α4-integrin expression selectively, and that effect was abrogated in *Drd3*-deficient B cells (Additional file 1: Fig. S7), thereby confirming that DRD3-signalling in B cells promotes α4-integrin expression. These results suggest that B-cell infiltration into the CNS may be impaired upon pMOG-induced EAE. Next, we addressed the possibility that DRD3 was regulating B-cell infiltration into the CNS upon pMOG-induced EAE. For this purpose, using congenic donor mice (*Cd45.1*^+*/*+^*/Cd45.2*^*−/−*^*/Drd3*^+*/*+^ and *Cd45.1*^*−/−*^*/Cd45.2*^+*/*+^*/Drd3*^*−/−*^ mice), we generated chimeric BM mice harbouring both *Drd3*-sufficient and *Drd3*-deficient B cells (Fig. 4D). In these animals, EAE was induced with pMOG, and the extent of B cells from different genotypes was compared in the CNS as well as in the spleen and peripheral blood. Interestingly, whereas the frequency of *Drd3*-sufficient B cells was increased in peripheral blood and the CNS upon EAE development, the frequency of *Drd3*-deficient B cells remained unaltered in all tissues evaluated upon EAE-development (Fig. 4E). Moreover, the analysis of absolute numbers of B cells revealed that in healthy conditions, *Drd3* deficiency resulted in a reduction in the number of B cells circulating through peripheral blood and the spleen, but with no significant effects in the CNS. Conversely, upon EAE development, *Drd3* deficiency induced a selective decrease in the number of B cells infiltrating the CNS and without effect in the number of B cells circulating through peripheral blood and the spleen (Additional file 1: Fig. S8). To explore whether the differences observed between *Drd3*-sufficient and *Drd3*-deficient B lymphocytes in the frequency and number of cells infiltrating the different tissues analysed were due to an effect of DRD3 in proliferation or cell death, we performed experiments in which B-cell activation was induced in *Drd3*^+*/*+^ or *Drd3*^*−/−*^ CD19^+^ cells, and the extent of proliferation and viability were determined. The results showed that *Drd3* deficiency resulted in a slight increase in B-cell proliferation and without effect in cell death (Fig. 4F, G), thereby ruling out the possibility that the reduction of cell number or frequency of *Drd3*-deficient B cells in the CNS was due to impaired proliferation or increased cell death. Thus, these results suggest that *Drd3* deficiency in B cells results in reduced α4-integrin expression and, consequently, in impaired B-cell infiltration into the CNS upon pMOG-induced EAE. Since our results associated DRD3 to the B-cell subset CD21^+^ CD23^+^ IgM^+^ (Additional file 1: Fig. S3B–D), which exerts anti-inflammatory activity in the CNS in the pMOG-induced EAE model (Fig. 2), we reasoned that *Drd3* deficiency should result in the accumulation of suppressive B cells in the spleen. Accordingly, we next sought to confirm the nature of cytokine produced by *Drd3*-deficient and *Drd3*-sufficient B cells retained in the periphery in this EAE model. For this purpose, at the peak of pMOG-induced EAE manifestation, CD45.1^+^ and CD45.2^+^ B cells were isolated from the spleen by cell-sorting, and the transcripts encoding for pro-inflammatory and anti-inflammatory cytokines were quantified by qRT-PCR. The results show that *Drd3* deficiency in B cells exacerbated *il10* transcription and reduced the expression of the mRNA encoding for GM-CSF (Fig. 4H). To confirm that DRD3-stimulation regulated the production of GM-CSF and IL-10 in B cells, we carried out in vitro experiments in which B cells were activated in the presence or the absence of dopamine, and the extent of cytokine production was assessed by flow cytometry. However, the results show that cytokine production was very low in these in vitro conditions, and no relevant differences were found (Additional file 1: Fig. S9). Taken together, these results indicate that DRD3 in B cells is required to induce full CNS-tropism in B cells with anti-inflammatory activity in a model of CNS autoimmunity that is independent of the APC-function of B cells.

### DRD3 favours the CXCR3 expression on B cells and their infiltration into the CNS in an EAE model that depends on the APC-function of B cells

**Fig. 5:**
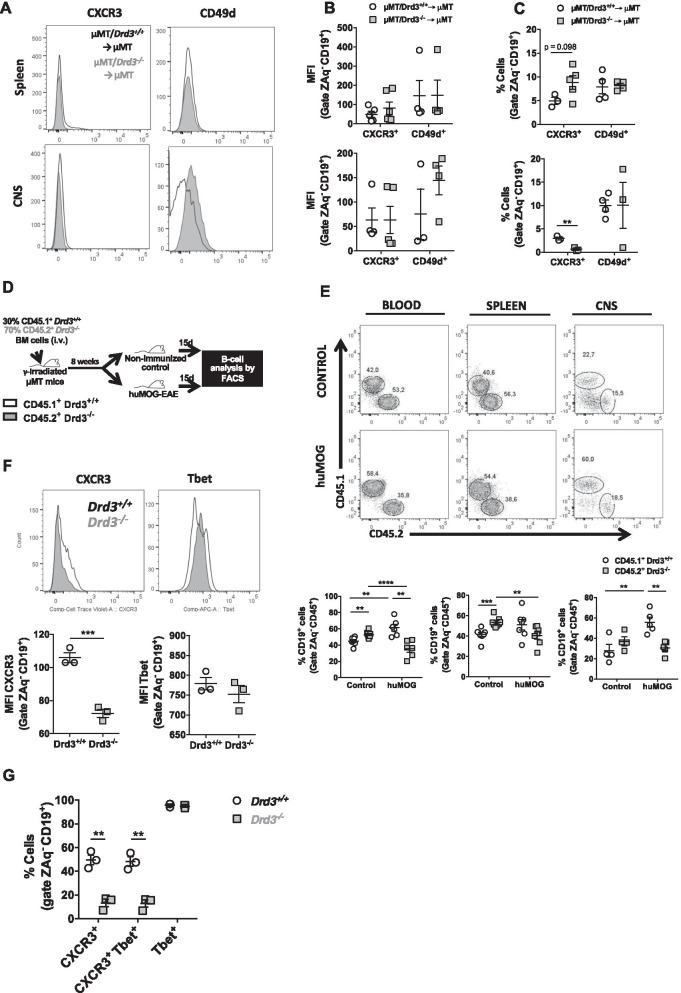
*Drd3* deficiency in B cells impairs the acquisition of CXCR3 and their infiltration into the CNS in an EAE model that depends on the APC-function of B cells. **A**–**C** BM chimeric mice harbouring *Drd3*-deficient (grey symbols) or *Drd3*-sufficient (white symbols) B cells were generated as described in Fig. 2A. Afterwards, EAE was induced in chimeric mice by immunization with huMOG in CFA followed by pertussis toxin injection. *n* = 3–6 mice per group. At the peak of disease severity (day 15 post-induction), mononuclear cells were isolated from the spleen and the CNS and the surface expression of CXCR3 and α4 integrin (CD49d) were analysed in the CD19^+^ population by flow cytometry. **A** Representative histograms for the expression of CXCR3 and CD49d in the CD19^+^ cells are shown. Quantification of the MFI (**B**) and frequency (**C**) associated to the surface expression of CXCR3 and CD49d in living (ZAq^−^) CD19^+^ cells isolated from the spleen (top panel) and CNS (bottom panel). **B**, **C** Each symbol represents data obtained from an individual mouse. The mean ± SEM are depicted. **, *p* < 0.01 by two-way ANOVA followed by Sidak’s post hoc test. **D**, **E** BM chimeric mice harbouring *Drd3*-deficient and *Drd3*-sufficient B cells were generated by the i.v. transfer of a 3:7 mixed BM from *Cd45.1*^+*/*+^*/Cd45.2*^*−/−*^*/Drd3*^+*/*+^ mice (white bars) and *Cd45.1*^*−/−*^*/Cd45.2*^+*/*+^*/Drd3*^*−/−*^ mice (grey bars) into γ-irradiated μMT recipient mice. Next, EAE was induced in chimeric mice by immunization with huMOG in CFA followed by pertussis toxin injection. **D** Schematic illustration of the experimental design. **E** At the peak of disease severity (day 15 post-induction), mononuclear cells were isolated from peripheral blood (left panels), the spleen (middle panels) and the CNS (right panels) and the frequency of total CD19^+^ B cells was analysed by flow cytometry. Top panels show representative dot-plots of CD45.1^+^ versus CD45.2^+^ cells in the CD19^+^ gate. Numbers indicate the percentage of cells in the corresponding region. Bottom panels show the percentage quantification. Each symbol represents data obtained from an individual mouse; *n* = 4–8 mice per group. The mean ± SEM are depicted. **, *p* < 0.01, ***, *p* < 0.001, ****, *p* < 0.0001 by two-way ANOVA followed by Sidak’s *post hoc* test. **F**, **G** Naïve B cells (CD19^+^ IgD^hi^ IgM^int^ CD11c^−^ TCRβ^−^) were isolated from the spleen of *Drd3*-deficient (grey histograms/symbols) or *Drd3*-sufficient (white histograms/symbols) mice by cell-sorting and incubated in vitro in the presence of anti-CD40, anti-IgM, IFNγ and the TLR9-ligand CpG. After 5 days, CXCR3 and Tbet expression were evaluated by flow cytometry. **F** Representative histograms of CXCR3 and Tbet expression in the CD19^+^ population are shown in top panels. Quantification of the mean fluorescence intensity (MFI) associated to CXCR3 (bottom left panel) and Tbet (bottom right panel) are shown. **G** Quantification of the percentage of CD19^+^ B cells positive for CXCR3, Tbet or both are shown. **F**, **G** Each symbol represents data obtained from an individual mouse; *n* = 3 mice per group. The mean ± SEM are depicted. **, *p* < 0.01; by two-way ANOVA followed by Sidak’s post hoc test

Finally, we attempted to determine the mechanism by which DRD3 in B cells exerts a pro-inflammatory effect in the development of CNS autoimmunity in a model dependent of the APC-function of B cells. Since our results show that disease manifestation was completely abrogated in mice harbouring *Drd3*-deficient B cells in this animal model, we next evaluated the role of DRD3 in the ability of B cells to act as APC. Accordingly, EAE was induced in µMT/*Drd3*^*−/−*^ → µMT and µMT/*Drd3*^+*/*+^ → µMT mice using huMOG, and at the peak of disease manifestation, the extent of class II MHC expression was evaluated in splenic B cells, nevertheless, no significant differences were found between both experimental groups (Additional file 1: Fig. S10A-B). Furthermore, the APC ability of *Drd3*^+*/*+^ and *Drd3*^*−/−*^ B cells was evaluated in vitro using huMOG-coupled beads as antigen and CD4^+^ T cells expressing a MOG-specific TCR as described before [53]. The results show that *Drd3* deficiency in B cells affected neither T-cell proliferation nor T-cell differentiation (Additional file 1: Fig. S10C-D). Similar conclusions were obtained when the APC-function of B cells was evaluated in vitro in the presence of dopamine at concentrations that stimulate DRD3 (Additional file 1: Fig. S11). Together, these results suggest that DRD3 was not relevant for APC-function of B cells upon huMOG-induced EAE. Since *Drd3 deficiency* in B cells affected the T-cell response only in the CNS, but not in the spleen (Fig. 3B–D) upon huMOG-induced EAE, we addressed the possibility that DRD3-signalling was affecting B-cell migration into the CNS in this model. Accordingly, EAE was induced with huMOG in µMT/*Drd3*^*−/−*^ → µMT and µMT/*Drd3*^+*/*+^ → µMT mice, and the extent of α4-integrin and CXCR3 expression was quantified in B cells infiltrating the CNS and the spleen (see the gating strategy in Figure S6). The results show that *Drd3* deficiency in B cells resulted in increased frequency of CXCR3 expression in the spleen and reduced CXCR3 expression in the CNS, without effect in α4-integrin expression in any tissue (Fig. 5A–C). Thus, these results suggest that B-cell infiltration into the CNS may be impaired by *Drd3* deficiency upon huMOG-induced EAE.

Next, we addressed the possibility that DRD3 was regulating B-cell infiltration into the CNS upon huMOG-induced EAE. For this purpose, using congenic donor mice (*Cd45.1*^*−/−*^*/Cd45.2*^+*/*+^*/Drd3*^*−/−*^ and *Cd45.1*^+*/*+^*/Cd45.2*^*−/−*^*/Drd3*^+*/*+^ mice), we generated chimeric BM mice harbouring both *Drd3*-sufficient and *Drd3*-deficient B cells (Fig. 5D). Because the transfer of *Drd3*^+*/*+^*-*to*-Drd3*^*−/−*^ BM at the 1:1 ratio resulted in decreased *Drd3*-deficient B cells in steady-state (Fig. 4D, E), in these experiments, we carried out the transfer of *Drd3*^+*/*+^*-*to*-Drd3*^*−/−*^ BM at 3:7 ratio. In these animals, EAE was induced with huMOG, and the extent of B cells from different genotypes was compared in the CNS as well as in the spleen and peripheral blood. The results show that frequency of *Drd3*-sufficient B cells was significantly increased in the CNS upon EAE development, whilst it was not changed in *Drd3*-deficient B cells (Fig. 5E). On the other hand, whereas the frequency of *Drd3*^*−/−*^ B cells was higher than *Drd3*^+*/*+^ B cells in the periphery upon steady-state, only *Drd3*^*−/−*^ B cells were reduced in frequency in peripheral blood and the spleen upon huMOG-induced EAE (Fig. 5E). Thus, these results suggest that *Drd3* deficiency in B cells results in altered CXCR3 expression and thereby impaired B-cell infiltration into the CNS upon huMOG-induced EAE. To gain a deeper mechanistic insight into the role of DRD3 in CXCR3 expression on B cells, we performed in vitro B-cell activation assay induced by anti-CD40, anti-IgM, IFNγ, and the TLR9-ligand CpG [60–62]. Since CXCR3 expression is dependent on the activity of the transcription factor Tbet in B cells [63], we activated naïve B cells isolated from *Drd3*^+*/*+^ and *Drd3*^*−/−*^ mice, and the extent of CXCR3 and Tbet expression was determined by flow cytometry. The results show that *Drd3* deficiency resulted in reduced frequency and density of CXCR3 expression without effect on Tbet expression (Fig. 5F, G), thus indicating that DRD3-signalling promotes CXCR3 expression downstream Tbet action. Together these results suggest that DRD3 promotes CXCR3 expression on pro-inflammatory B cells and the consequent infiltration into the CNS upon huMOG- induced EAE.

## Discussion

Our findings demonstrated an important role of dopaminergic stimulation mediated by DRD3 in promoting the infiltration of pro-inflammatory and anti-inflammatory B cells in the CNS in two different animal models of CNS autoimmunity.

Interestingly our data show that the frequency of DRD3^+^ B cells was selectively increased in the CNS upon huMOG-induced EAE. Further phenotypical analysis shows that the only B-cell population expressing DRD3 in the CNS in this EAE model was the CD20^+^ MHC-II^+^ subset. A previous study shows that this B-cell population exerts a fundamental function as APC in the CNS upon huMOG-induced EAE [38]. Nevertheless, it is important to consider that MHC-II expression in B cells is also required for the production of T-cell-dependent autoantibodies [64–66]. According to the fundamental function of CD20^+^ MHC-II^+^ B cells in CNS autoimmunity and to the important role of DRD3 favouring the infiltration of these B cells in the CNS, *Drd3* deficiency in B cells abrogated disease manifestation completely and strongly reduced the frequency of inflammatory CD4^+^ T cells in this animal model (Fig. 3).

Our mechanistic analysis showed that DRD3 was not necessary for APC-function of B cells but was required to induce an efficient CNS-tropism in this B-cell subset. This DRD3-induced CNS-tropism was mediated by an upregulation of CXCR3 expression. According to these findings, a recent study showed that B cells expressing CXCR3 were enriched in the CNS of MS patients [63]. Moreover, the treatment of MS patients with a clinically effective drug, natalizumab, which avoids lymphocytes infiltration into the CNS, decreased the accumulation of CXCR3^+^ B cells into the CNS [63]. Interestingly, IFNγ might induce the expression of the transcription factor Tbet, which promotes the expression of CXCR3 in lymphocytes, including B cells. This chemokine receptor confers responsiveness to the chemokines CXCL9, CXCL10, and CXCL11, which are produced at high levels in sites of inflammation in response to IFNγ [67]. Our results showed that *Drd3* deficiency in B cells attenuated the expression of CXCR3 without effects in the level of Tbet expression, indicating that DRD3 in B cells favours CXCR3 expression downstream Tbet.

Using a model of CNS autoimmunity in which the APC-function of B cells is irrelevant, we found another important regulatory effect of DRD3 in the control of B-cell function. Our data show that *Drd3* deficiency in B cells resulted in exacerbated disease manifestation and increased frequency of CD4^+^ T cells producing GM-CSF in the CNS upon pMOG-induced EAE. Of note, it has been demonstrated that GM-CSF is the most pro-inflammatory cytokine produced by CD4^+^ T cells in the CNS. In contrast to IL-17 and IFNγ, GM-CSF is essential to promote EAE manifestation [57]. Thereby, our results suggested that DRD3 stimulation favoured the function of B cells with anti-inflammatory activity in the CNS. In this regard, three different subsets of B cells with immunosuppressive function have been described so far, including CD1d^+^ CD5^+^ (also called B10) [35, 68], CD138^+^ CD44^+^ [69] and CD21^+^ CD23^+^ IgM^+^ B cells [55]. All of these subsets of regulatory B cells exert immunosuppressive effects, attenuating the T-cell-mediated inflammation, which is mediated by IL-10. Interestingly, we observed that CD21^+^ CD23^+^ IgM^+^ was the only subset of regulatory B cells detectable in the CNS upon pMOG-induced EAE, whilst CD1d^+^ CD5^+^ and CD138^+^ CD44^+^ subsets were barely detectable in these conditions. In addition, CD21^+^ CD23^+^ IgM^+^ B cells were the only subset of regulatory B cells expressing detectable levels of DRD3 on the cell surface upon pMOG-induced EAE. Further analysis showed that DRD3 was necessary to induce the upregulation of α4-integrin (CD49d) on the cell surface and the subsequent B-cell infiltration into the CNS upon pMOG-induced EAE. Importantly, the α4-integrin together β1-integrin (CD29) form a heterodimeric complex on the lymphocyte surface that recognizes its ligand, the vascular cell adhesion protein 1 (VCAM-1) expressed in the blood–brain barrier, allowing the lymphocyte infiltration into the CNS [70, 71]. Notably, the genetic deficiency of α4-integrin restricted to the CD19^+^ B cells resulted in exacerbated CNS autoimmunity in a model of pMOG-induced EAE [72], indicating the high relevance of α4-integrin in the infiltration of regulatory B cells into the CNS in this EAE model. Accordingly, our results showed that *Drd3* deficiency in B cells not only resulted in impaired α4-integrin expression and reduced B-cell infiltration into the CNS, but also in the accumulation of B cells displaying immunosuppressive potential (higher *il10* and lower *cfs2* transcription) in the periphery.

Although the pMOG-induced EAE model represents a proper experimental system to study B cells' regulatory role irrespective of their APC-function in the CNS, the huMOG-induced EAE model mimics better the B-cell participation in MS. According to this notion, it has been shown that natalizumab, an anti-α4-integrin monoclonal antibody, exerts an efficient therapeutic effect reducing disease manifestation in relapsing–remitting MS patients [59, 73]. Agree with this observation in human patients, mice harbouring α4-integrin-deficient B cells display a significant reduction in disease manifestation upon huMOG-induced EAE model [74]. In contrast to the results observed in MS patients, the genetic deficiency of α4-integrin in B cells resulted in exacerbated disease manifestation in a pMOG-induced EAE model [72]. Another aspect supporting the idea that huMOG-induced EAE is a proper experimental system to study B-cell participation in MS is based on CXCR3. According to the relevant role of CXCR3 in the infiltration of inflammatory B cells observed here upon huMOG-induced EAE, CXCR3 has been found highly expressed on B cells obtained from the CSF, meninges, and brain of MS patients [59, 63]. In addition, the treatment of patients with natalizumab, which exerts an efficient therapeutic effect, reduced the accumulation of CXCR3^+^ B cells into the CNS [63].

Dopaminergic signalling has been previously involved in the control of migration of other cells of the immune system. A recent study described that DRD4-stimulation induced an upregulation of CCR5 in human macrophages and thus increasing their migratory ability to infiltrate the brain [74]. Conversely, signalling through DRD1 was identified as a negative regulator of CCR5 expression [74]. According to these results, the inhibition of dopamine synthesis induced by α-methylparatyrosine resulted in a decreased recruitment of peripheral monocytes into the nigrostriatal pathway in a mouse model of Parkinson’s disease [75]. In addition, addressing the role of dopamine in the migration of lymphocytes, Watanabe et al. have provided pharmacologic evidence suggesting that DRD3-signalling favours CD8^+^ T-cell migration in response to CCL19, CCL21, and CXCL12 [76]. According to the role of CCR7 (receptor for CCL19 and CCL21) in the recirculation of naïve T cells into the lymph nodes throughout the body, the systemic antagonism of DRD3 decreased the recruitment of naïve CD8^+^ T-cell into the lymph nodes [76]. Moreover, another work provided pharmacologic evidence supporting the notion that DRD5-signalling reduces CCR4 expression in Treg and thereby, attenuates the recruitment of these cells to CCL22 [77]. In addition, we recently found that DRD3-signalling down-regulates CCR9 expression on Treg, thus limiting their recruitment into the inflamed gut mucosa and consequently exacerbating inflammatory colitis [15]. Here, we provide in vivo evidence using genetic approaches demonstrating that DRD3 in B cells mediates the upregulation of CXCR3 and α4-integrin, thus affecting the recruitment of important B-cell subsets into the CNS upon the development of CNS autoimmunity.

Interestingly, we recently found that DRD5 might be assembled with CCR9 in inflammatory T cells, conforming a CCR9:DRD5 heteromeric receptor that drives the recruitment of these cells into the gut mucosa upon inflammation [78]. The stimulation of the CCR9:DRD5 heteromer exerts a biological function different from those downstream functions triggered by the isolated forms of CCR9 or DRD5, thus constituting a new cell surface sensor. This phenomenon raises the possibility that under certain circumstances, DRD3 could be part of a heteromeric receptor on B cells, which might explain why, in some cases, there were disparities between the in vitro DRD3-stimulation experiments and the in vivo DRD3 deficiency experiments. Nevertheless, to confirm this possibility, further experimental research is necessary.

Regarding the role of dopaminergic signalling in lymphocytes inside the CNS during EAE, evidence suggests that high dopamine levels attenuate the inflammatory potential of these cells [2]. Consistent with this idea, the systemic administration of a monoaminoxidase inhibitor, which induces a significant increase of dopamine levels in the brain and spinal cord, dampens the development of EAE manifestation in mice [79]. Of note, this effect was not due to an alteration in the extent of T-cell infiltration in the CNS [79]. Conversely, the reduction of striatal dopamine levels exerted by the treatment of mice with MPTP prior EAE induction results in exacerbated disease manifestation [22].

Considering the anti-inflammatory effect reported for DRD2 stimulation [1, 2], the expression of DRD2 in B cells (Additional file 1: Fig. S5) [48], and the dominant action of DRD2-signalling over DRD3-signalling [49], it is possible to speculate that CNS areas maintaining high dopamine levels (≥ 10^–6^ M), including the hippocampus, paraventricular nucleus, striatum and retina [23], would promote an anti-inflammatory effect mediated by DRD2 on B cells under homeostatic conditions. However, CNS areas that involve a reduction of dopamine levels upon EAE in rodents, such as the spinal cord and the striatum [23], would promote the selective DRD3 stimulation on B cells.

In this regard, DRD3-stimulation in Breg infiltrating the striatum and spinal cord would promote a higher anti-inflammatory function. Since no alterations of APC-function of inflammatory B cells were found due to *Drd3* deficiency, it is tempting to speculate that the selective DRD3-stimulation in B cells with APC-function infiltrating the striatum and spinal cord would not affect the inflammatory potential of these cells. However, the DRD3-stimulation on B cells with APC-function in peripheral tissues where dopamine levels are low (10^–9^–10^–7^ M), such as plasma, bone marrow, or spleen [23], would promote a higher infiltration of these inflammatory cells into the CNS (Figs. 3, 5) upon EAE development, thus exacerbating the disease severity.

## Conclusions

Our findings demonstrate here for the first time how dopaminergic stimulation in B cells exerts an important regulation in the development of CNS autoimmunity. First, through DRD3, dopamine favours the CNS-tropism in a pro-inflammatory B-cell subset with APC-function, thus contributing to the re-stimulation of encephalitogenic effector CD4^+^ T cells and thereby reinforcing CNS autoimmunity. Secondly, when B cells with APC-function are negligible, DRD3-signalling promotes CNS-homing of B cells with anti-inflammatory features and, consequently, dampens the T-cell-mediated autoimmunity.

## Supplementary Information

**Additional file 1: Figure S1.** B-cell infiltration into the CNS correlates with the EAE clinical score (associated to Fig. 1). EAE was induced in C57BL/6 mice (*n* = 6). A group of control mice were treated only with PBS (*n* = 2). At day 15 post-induction, mice were killed and the extent of B cells infiltrating the brain was analysed by immunofluorescence. (A) Representative images of immunofluorescence for CD19 in mice displaying clinical score 0 (left panel, healthy control), 2 (middle panel, EAE) and 3 (right panel, EAE). Arrow heads showing some CD19 ^+^ cells in the tissue. Bar, 200 µm. Inserts in the down-left corner show some B cells in higher magnification. (B) The number of CD19 ^+^ cells per area was quantified in the cortex (*n* = 3 slides per mouse) and a correlation analysis was performed with the clinical score. The R2 and *p* value were calculated with Pearson’s correlation coefficient.** Figure S2.** Analysis of DRD3 expression in B cells upon EAE development (associated to Fig. 1). EAE was induced in wild-type C57BL/6 mice by immunization with pMOG_35–55_ or huMOG in CFA followed by pertussis toxin injection. At the peak of disease severity, mononuclear cells were isolated from the spleen, draining lymph nodes (dLN) and central nervous system (CNS) and DRD3 expression was evaluated in the CD19^ +^ population by flow cytometry. (A) Gating strategy. Numbers indicate the percentage of cells inside the selected region. (B) Representative dot plots showing the gate of CD19 ^+^ cells from each tissue selected for the analysis of DRD3 expression. Numbers indicate the percentage of CD19 ^+^ cells. (C) Quantification of the mean fluorescence intensity (MFI) associated to DRD3 immunostaining. Left panels show representative histograms. Unspecific (black lined) histograms corresponds to controls in which anti-DRD3 antibody was pre-incubated with the antigenic peptide (used as immunogen to develop the antibody) to avoid specific binding on the cell surface. Right panels show the quantification of the MFI in the CD19 ^+^ gate. Each symbol represents data obtained from an individual mouse; *n* = 2–3 mice per group. The mean ± SEM are depicted. ***, *p* < 0.001; ****, *p* < 0.0001 by one-way ANOVA followed by Tukey’s post hoc test. **Figure S3.** Distribution and DRD3 expression of B-cell subpopulations upon EAE development (associated to figure 1). EAE was induced in C57BL/6 mice by immunization with pMOG_35–55_ or huMOG in CFA followed by pertussis toxin injection. Disease severity was evaluated as clinical score during the time-course of the disease development. At maximum disease severity (score 3), mononuclear cells were isolated from Spleen, draining lymph node (dLN) and Central nervous system (CNS). (A) Representative dot plots showing the frequency of B cells (CD19^+^ cells) observed in the analysed tissues. (B) Representative dot plots indicating different B-cell subpopulations analysed from the CD19^+^ gate. (C and D) the frequency (C) and the percentage of DRD3 expression (D) in different subpopulations of CD19^+^ B cells were evaluated. Data representative from one out of three independent experiments are shown. Values represent mean ± SEM from 3-5 mice per group. *, *p*<0.05; **, *p*<0.01; ***, *p*<*p*.001; ****, *p*<0.0001 by one-way ANOVA followed by Tukey’s post hoc test. **Figure S4.** Analysis of leukocyte populations in bone marrow chimeric mice harbouring *Drd3* deficiency restricted to B cells (associated to figure 2 and 3). μMT recipient mice were γ-irradiated with 1100 rads and 24 h later reconstituted with a bone marrow mixture (107 total cells per mouse) conformed by 80% obtained from μMT mice and 20% obtained from *Drd3*^*+/+*^ or 20% *Drd3*^*-/-*^ mice (see an scheme in figure 2A). Eight weeks after BM-transfer, leukocyte populations were analysed in peripheral blood by flow cytometry. Quantification of the absolute number of *Drd3*-sufficient and *Drd3*-deficient leukocyte populations is shown. Each symbol represents data obtained from an individual mouse; *n* = 4-5 mice per group. The mean ± SEM are depicted. Not significant differences were detected between both genotypes. **Figure S5.** *Drd3* deficiency does not affect the expression of other components of the dopaminergic system in B cells (associated to figure 1). The expression of tyrosine hydroxylase (TH, left panels), dopamine receptor D2 (DRD2, middle panels) and dopamine receptor D5 (DRD5, right panels) was analysed in the CD19^*+*^ population in *Drd3*-sufficient (black lines) and *Drd3*-deficient (green lines) splenic B cells by flow cytometry. TH immunostaining was performed in permeabilized cells whilst DRD2 and DRD5 immunostaining was carried out in non-permeabilized cells. Top panels show representative histograms. The grey histograms represent negative controls of immunostaining: an isotype-matched control in the case of TH (left panel), or the pre-treatment of the anti-DRD2 or anti-DRD5 antibodies with the specific antigenic peptide recognized by the respective antibody (middle and right panels). Bottom panels show the quantification of the MFI associated to the immunostaining normalized by the MFI associated to negative control (In-fold). The dotted line shows in-fold = 1, which indicates no expression. Each symbol represents data obtained from an individual mouse; *n* = 4 mice per group. The mean ± SEM are depicted. Not significant differences were detected between both genotypes. **Figure S6.** Gating strategy to analyse surface expression of homing molecules in B cells (associated to figure 4 and 5). (A) Representative dot-plots showing the gating strategy to analyse surface expression of homing molecules in splenic B cells isolated from *Drd3*-sufficient mice. Numbers indicate the percentage of cells inside the selected region. (B) Representative dot plots showing the analysis of expression of homing molecules in B cells selected as in (A). Left panel shows the fluorescence associated to CXCR3 (BV421) and CD49d (PE) immunostaining. Middle panel shows the FMO for the BV421 channel, whilst right panel shows the FMO for the PE channel. Numbers indicate the percentage of cells inside the corresponding quadrant. (C) Representative histograms showing the fluorescence associated to CXCR3 immunostaining (left panel) and CD49d immunostaining (right panel). Black lined histograms represent fluorescence associated to FMO, whilst red lined histograms represent fluorescence associated to CXCR3 and CD49d immunostaining, respectively. **Figure S7.** *Drd3*-signalling increases CD49d expression in B cells (associated to figure 4). CD19^+^ IgM^int^ CD11c^-^ TCRβ^-^ cells were isolated by cell-sorting and then in vitro activated in the absence (control) or in the presence of 500 nM Dopamine (Dopamine) for 48h. Afterwards, the extent of surface expression of CXCR3 (left panel) or CD49d (right panel) was analysed by flow cytometry. (A) Representative histograms. (B) Quantification. Values are the mean fluorescence intensity (MFI) associated to the immunostaining of CXCR3 (left panel) or CD49d (right panel) of ZAq^-^ CD19^+^ cells. Each symbol represents data obtained from an individual mouse; *n* = 3-6 mice per group. The mean ± SEM are depicted. *, *p*<0.05 by two-way ANOVA followed by Sidak’s post hoc test. **Figure S8.** Drd3 deficiency results in reduced number of B cells under homeostasis or upon EAE induction (associated to figure 4). Chimeric mice were generated and treated as shown in figure 4D and, at the peak of disease severity (day 15 post-induction), mononuclear cells were isolated from peripheral blood (left panel), the spleen (middle panel) and the CNS (right panel) and the absolute number of CD19+ B cells was quantified by flow cytometry. Each symbol represents data obtained from an individual mouse; *n* = 4 mice per group. The mean ± SEM are depicted. *, *p*<0.05; **. P<0.01; ***, *p*<0.001, ****, *p*<0.0001 by two-way ANOVA followed by Sidak’s post hoc test. **Figure S9.** Analysis of cytokine production by activated B cells in the presence of dopamine (associated to figure 4). *Drd3*-sufficient or *Drd3*-deficient CD19^+^ IgM^int^ CD11c^-^ TCRβ^-^ cells were isolated from the spleen by cell-sorting and then in vitro activated in the absence (control) or in the presence of 500 nM Dopamine (Dopamine) for 5 d. Afterwards, cells were re-stimulated with PMA and ionomycin in the presence of brefeldin A for 4 h and the extent of IL-10, IL-6 and GM-CSF production was assessed by intracellular cytokine immunostaining and analysed by flow cytometry. (A) Representative dot-plots are shown. (B) Quantification. Values are the percentage of cells producing the corresponding cytokine in the ZAq^-^ CD19^+^ gate. Each symbol represents data obtained from an individual mouse; *n* = 5-7 mice per group. The mean ± SEM are depicted. *, *p*<0.05 by two-way ANOVA followed by Sidak’s post hoc test. **Figure S10.** Drd3 deficiency in B cells does not impair antigen-presentation to T cells (associated to figures 4 and 5). (A and B) MHC-II expression was evaluated by Flow cytometry analysis in splenic CD19^+^ B cells obtained from μMT/*Drd3*^*+/+*^ or μMT/*Drd3*^*-/-*^ chimeric mice immunized with (A) pMOG or (B) huMOG. (C-D) In vitro antigen-presentation assays. 2D2 CD4^+^ T cells, which express the transgenic TCR specific for recognizing the peptide pMOG_35-55_ on IA^b^, were loaded with the fluorescent probe Cell Trace Violet (CTV). B cells were pulsed with (C) pMOG-beads or (D) huMOG-beads overnight and then co-cultured with CTV-loaded 2D2 CD4^+^ T cells. After 5 days, proliferation as well as cytokine production were determined by flow cytometry in living (ZAq^-^) CD4^+^ T cells. Each symbol represents data obtained from an individual mouse; *n* = 3-9 mice per group. The mean ± SEM are depicted. Not significant differences were detected between both genotypes. **Figure S11.** (associated to figure 5). *Drd3*-signalling in B cells acting as APC does not affect the extent of cytokine production by T cells. *Drd3*-sufficient or *Drd3*-deficient CD19^+^ IgM^int^ CD11c^-^ TCRβ^-^ cells were isolated by cell-sorting and then in vitro pulsed with the antigen in the absence (control) or in the presence of 500 nM Dopamine (Dopamine) for 2h. Afterwards, B cells were washed and co-cultured with 2D2 CD4^+^ T cells, which were previously loaded with the fluorescent probe Cell Trace Violet (CTV). After 5 days, cells were re-stimulated with PMA and ionomycin for 5 h in the presence of brefeldin A, and the extent of IL-17 (A), GM-CSF (B) and IFN-γ (C) production was determined by intracellular cytokine staining in the living (ZAq^-^) CD4^+^ population. The expression of Foxp3 in the living (ZAq^-^) CD4^+^ T-cell population was assessed by intracellular immunostaining followed by flow cytometry (D). The extent of proliferation of living (ZAq^-^) CD4^+^ T cells was evaluated as the percentage of cells displaying dilution of the fluorescence associated to CTV by flow cytometry (E). Top panel shows representative histograms; bottom panel shows the quantification. (A-E) Each symbol represents data obtained from an individual mouse; *n* = 4-5 mice per group. The mean ± SEM are depicted. Not significant differences were detected between genotypes or treatments.

## Data Availability

The datasets used and/or analysed during the current study are available from the corresponding author on reasonable request.

## Abbreviations

ACK: Ammonium-chloride-potassium
Ab: Antibody
APC: Antigen-presenting cells
BCR: B-cell receptor
BM: Bone marrow
CNS: Central nervous system
CD*n*: Cluster of differentiation *n*
DCs: Dendritic cells
dLN: Draining lymph nodes
DR: Dopamine receptor
DR D*n*: DRD*n*
EAE: Experimental autoimmune encephalomyelitis
huMOG: Human MOG
MFI: Mean fluorescence intensity
MOG: Myelin oligodendrocyte glycoprotein
MS: Multiple sclerosis
pMOG_35-55_: Peptide MOG_35-55_
PBMC: Peripheral blood mononuclear cells
Rag1: Recombination activating gene 1
Tregs: Regulatory T cells
Tbet: T-box transcription factor encoded by TBX21
Th*n*: T helper *n*
VCAM-1: Vascular cell adhesion molecule 1
