# Supplementary material for: Dopaminergic stimulation leads B-cell infiltration into the central nervous system upon autoimmunity
Source: J Neuroinflammation. 2021 Dec 17;18:292. doi: 10.1186/s12974-021-02338-1 (PMC8680379; doi:10.1186/s12974-021-02338-1)
Supplement: Supplementary file 1 — Additional file 1: Figure S1. B-cell infiltration into the CNS correlates with the EAE clinical score (associated to Fig. 1). EAE was induced in C57BL/6 mice (n = 6). A group of control mice were treated only with PBS (n = 2). At day 15 post-induction, mice were killed and the extent of B cells infiltrating the brain was analysed by immunofluorescence. (A) Representative images of immunofluorescence for CD19 in mice displaying clinical score 0 (left panel, healthy control), 2 (middle panel, EAE) and 3 (right panel, EAE). Arrow heads showing some CD19 + cells in the tissue. Bar, 200 µm. Inserts in the down-left corner show some B cells in higher magnification. (B) The number of CD19 + cells per area was quantified in the cortex (n = 3 slides per mouse) and a correlation analysis was performed with the clinical score. The R2 and p value were calculated with Pearson’s correlation coefficient. Figure S2. Analysis of DRD3 expression in B cells upon EAE development (associated to Fig. 1). EAE was induced in wild-type C57BL/6 mice by immunization with pMOG35–55 or huMOG in CFA followed by pertussis toxin injection. At the peak of disease severity, mononuclear cells were isolated from the spleen, draining lymph nodes (dLN) and central nervous system (CNS) and DRD3 expression was evaluated in the CD19 + population by flow cytometry. (A) Gating strategy. Numbers indicate the percentage of cells inside the selected region. (B) Representative dot plots showing the gate of CD19 + cells from each tissue selected for the analysis of DRD3 expression. Numbers indicate the percentage of CD19 + cells. (C) Quantification of the mean fluorescence intensity (MFI) associated to DRD3 immunostaining. Left panels show representative histograms. Unspecific (black lined) histograms corresponds to controls in which anti-DRD3 antibody was pre-incubated with the antigenic peptide (used as immunogen to develop the antibody) to avoid specific binding on the cell surface. Right panels show the quant [file 12974_2021_2338_MOESM1_ESM.pdf]

Additional file 1

Dopaminergic stimulation leads B-cell infiltration into the central nervous system upon autoimmunity

Carolina Prado, Francisco Osorio-Barrios, Paulina Falcón, Alexandra Espinoza, Juan José Saez, María Isabel Yuseff, and Rodrigo Pacheco.

Figure S1. Prado et al.

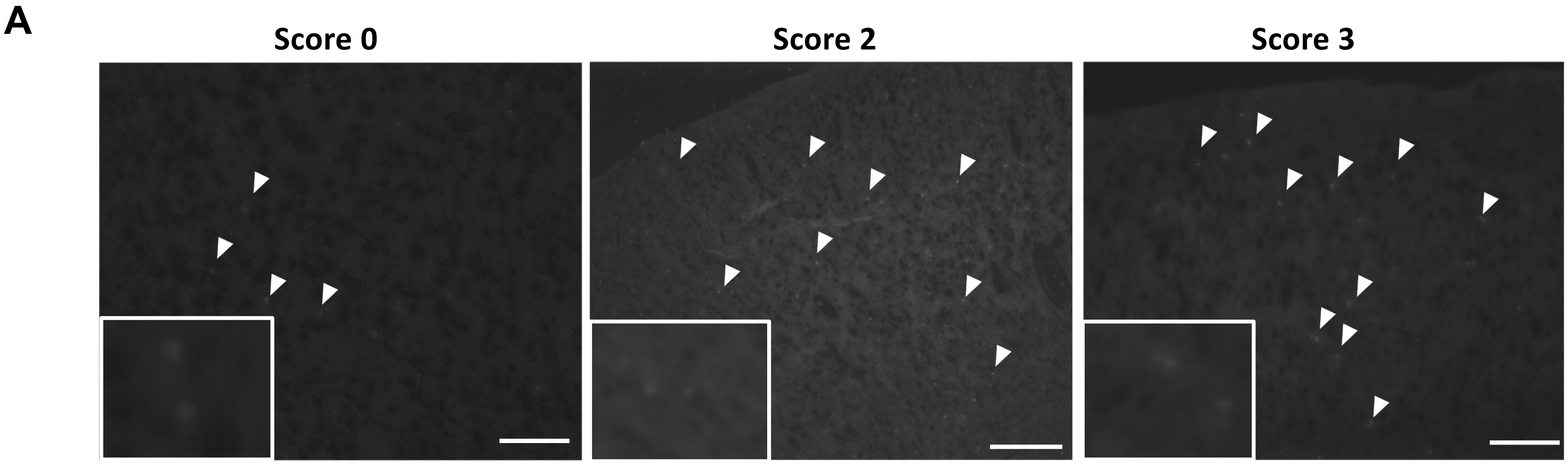

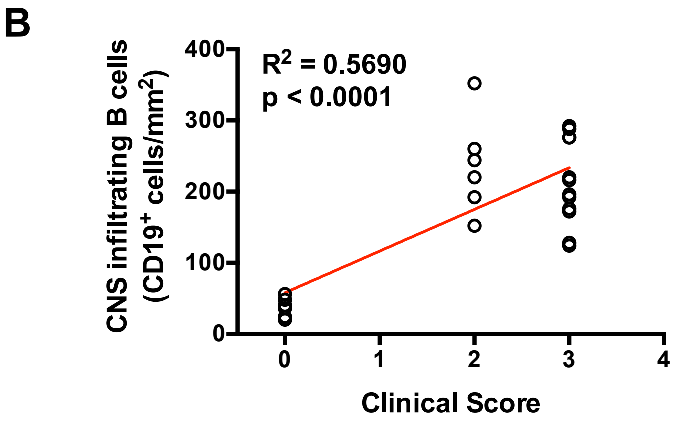

Figure S1. B-cell infiltration into the CNS correlates with the EAE clinical score (associated to figure 1). EAE was induced in C57BL/6 mice (n = 6). A group of control mice were treated only with PBS (n=2). At day 15 post-induction, mice were sacrificed and the extent of B-cells infiltrating the brain was analysed by immunofluorescence. (**A**) Representative images of immunofluorescence for CD19 in mice displaying clinical score 0 (left panel, healthy control), 2 (middle panel, EAE) and 3 (right panel, EAE). Arrow heads showing some $CD19^+$ cells in the tissue. Bar, 200 μm. Inserts in the down-left corner show some B cells in higher magnification. (**B**) The number of $CD19^+$ cells per area was quantified in the cortex (n = 3 slides per mouse) and a correlation analysis was performed with the clinical score. The R^2 and p value were calculated with Pearson's correlation coefficient.

Figure S2. Prado et al.

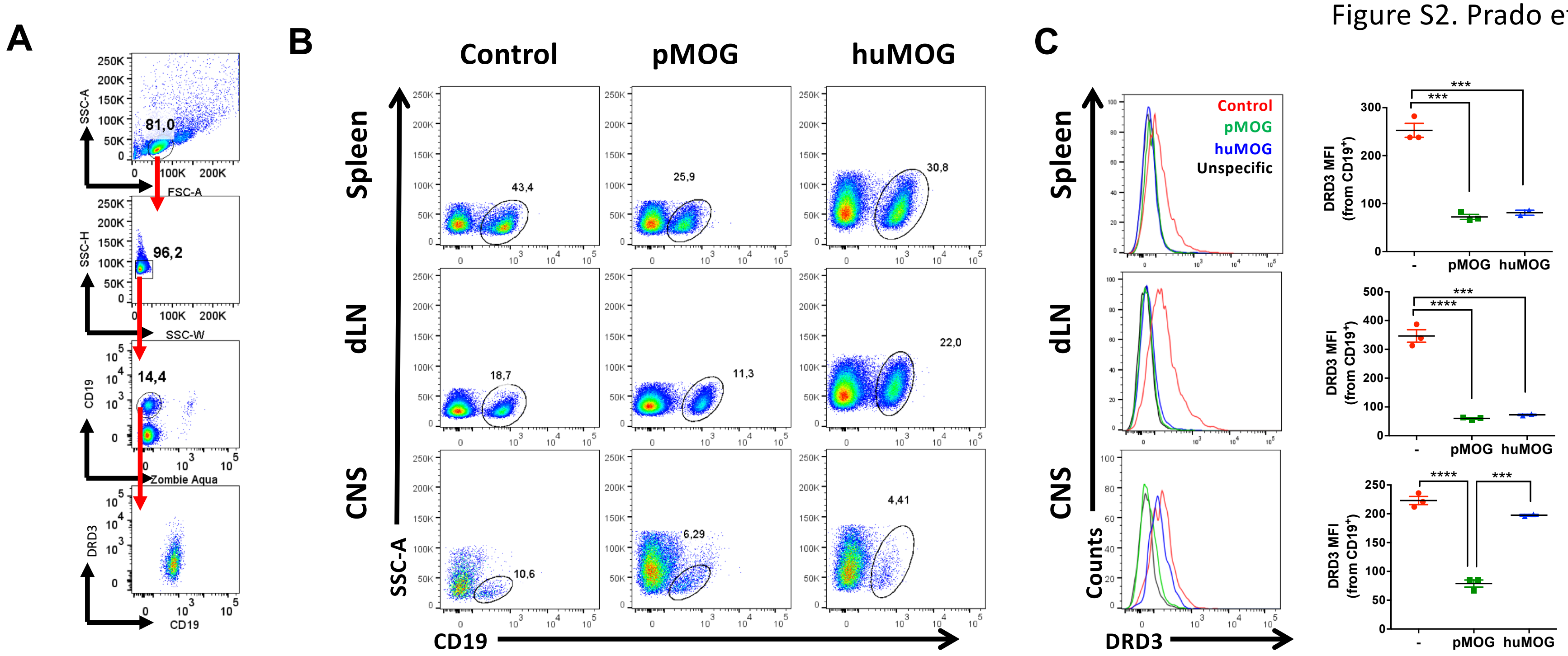

Figure S2. Analysis of DRD3 expression in B-cells upon EAE development (associated to figure 1). EAE was induced in wild-type C57BL/6 mice by immunization with pMOG$_{35\text{-}55}$ or huMOG in CFA followed by pertussis toxin injection. At the peak of disease severity, mononuclear cells were isolated from the spleen, draining lymph nodes (dLN) and central nervous system (CNS) and DRD3 expression was evaluated in the CD19$^+$ population by flow cytometry. (**A**) Gating strategy. Numbers indicate the percentage of cells inside the selected region. (**B**) Representative dot plots showing the gate of CD19$^+$ cells from each tissue selected for the analysis of DRD3 expression. Numbers indicate the percentage of CD19$^+$ cells. (**C**) Quantification of the mean fluorescence intensity (MFI) associated to DRD3 immunostaining. Left panels show representative histograms. Unspecific (black lined) histograms corresponds to controls in which anti-DRD3 antibody was pre-incubated with the antigenic peptide (used as immunogen to develop the antibody) to avoid specific binding on the cell surface. Right panels show the quantification of the MFI in the CD19$^+$ gate. Each symbol represents data obtained from an individual mouse; n = 2-3 mice per group. The mean ± SEM are depicted. ***, p<0.001; ****, p<0.0001 by one-way ANOVA followed by Tukey's *post-hoc* test.

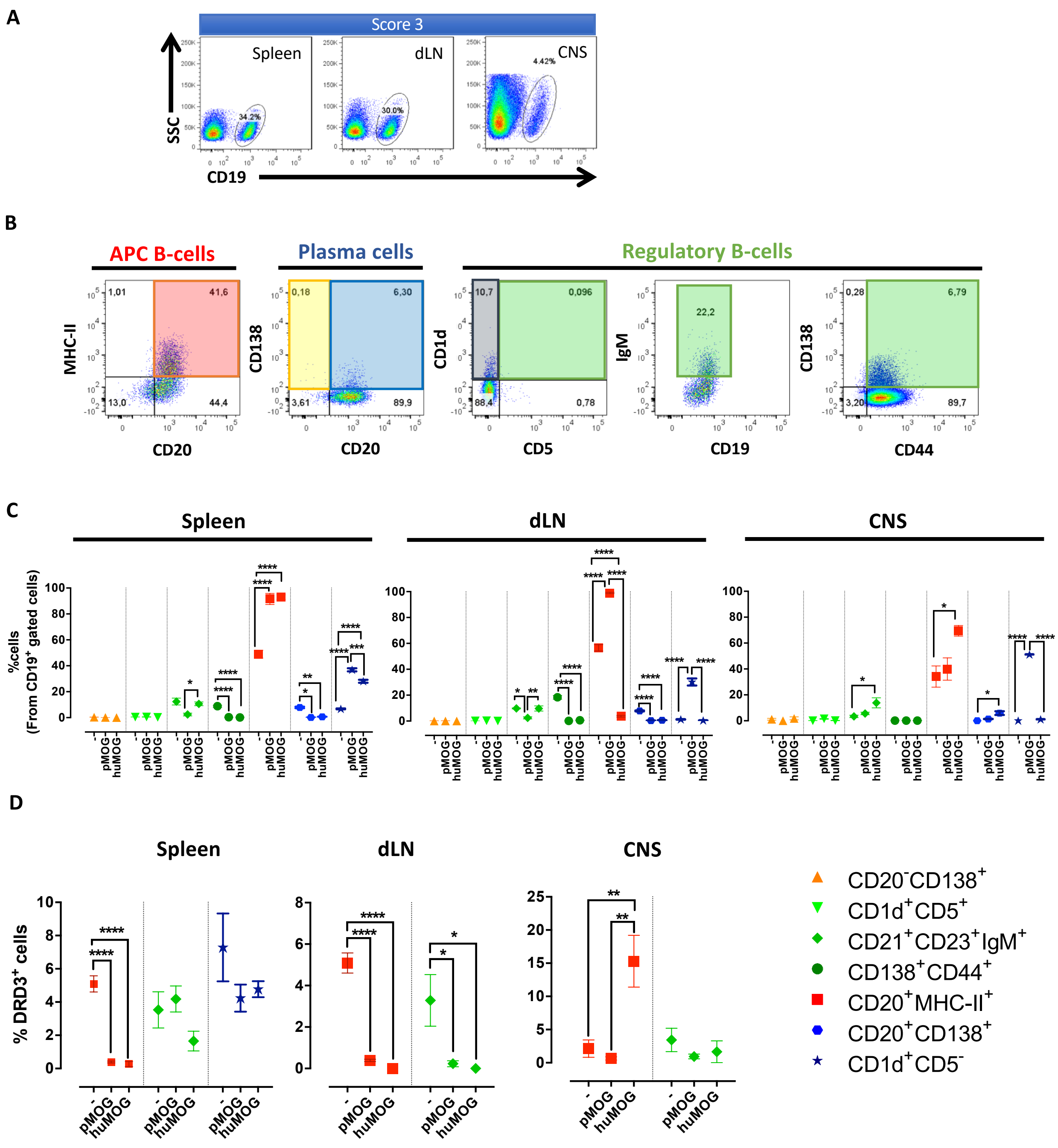

Figure S3. Distribution and DRD3 expression of B cell sub-populations upon EAE development (associated to figure 1). EAE was induced in C57BL/6 mice by immunization with pMOG$_{35-55}$ or huMOG in CFA followed by pertussis toxin injection. Disease severity was evaluated as clinical score during the time-course of the disease development. At maximum disease severity (score 3), mononuclear cells were isolated from Spleen, draining lymph node (dLN) and Central nervous system (CNS). (**A**) Representative dot plots showing the frequency of B-cells (CD19$^+$ cells) observed in the analysed tissues. (**B**) Representative dot plots indicating different B-cells subpopulations analyzed from the CD19$^+$ gate. (**C** and **D**) the frequency (**C**) and the percentage of DRD3 expression (**D**) in different subpopulations of CD19$^+$ B-cells were evaluated. Data representative from one out of three independent experiments is shown. Values represent mean ± SEM from 3-5 mice per group. *, p<0.05; **, p<0.01; ***, p<p.001; ****, p<0.0001 by one-way ANOVA followed by Tukey's *posthoc* test.

Figure S4. Prado et al.

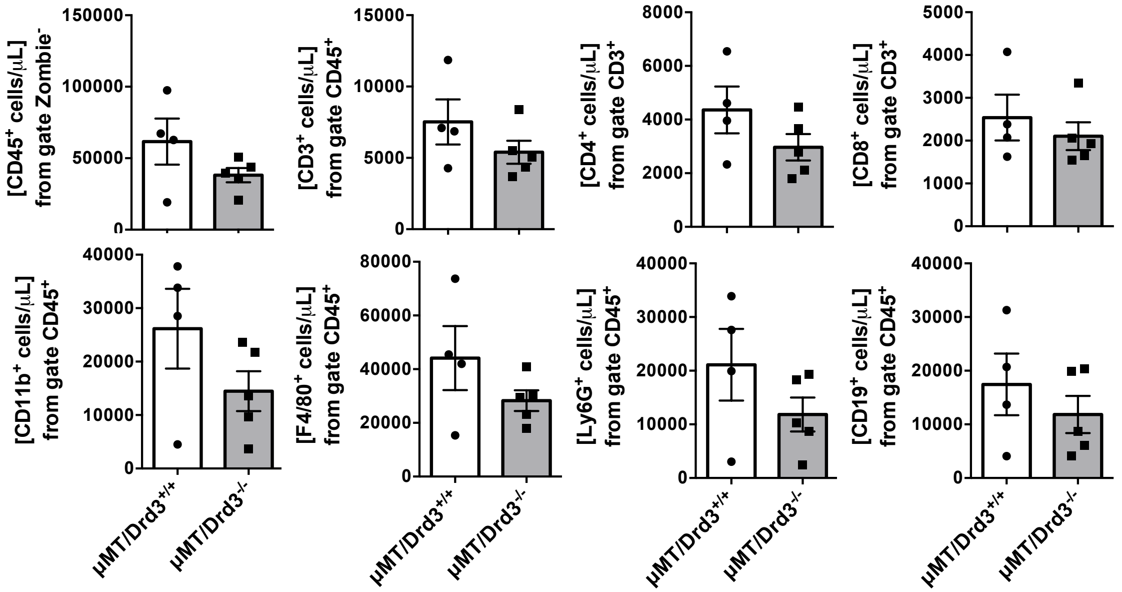

Figure S4. Analysis of leukocyte populations in bone-marrow chimeric mice harbouring *Drd3* deficiency restricted to B-cells (associated to figure 2 and 3). µMT recipient mice were γ-irradiated with 1100 rads and 24 h later reconstituted with a bone-marrow mixture (10^7 total cells per mouse) conformed by 80% obtained from µMT mice and 20% obtained from *Drd3*$^{+/+}$ or 20% *Drd3*$^{-/-}$ mice (see an scheme in figure 2A). Eight weeks after BM transfer, leukocyte populations were analysed in peripheral blood by flow cytometry. Quantification of the absolute number of *Drd3*-sufficient and *Drd3*-deficient leukocyte populations is shown. Each symbol represents data obtained from an individual mouse; n = 4-5 mice per group. The mean ± SEM are depicted. Not significant differences were detected between both genotypes.

Figure S5. Prado et al.

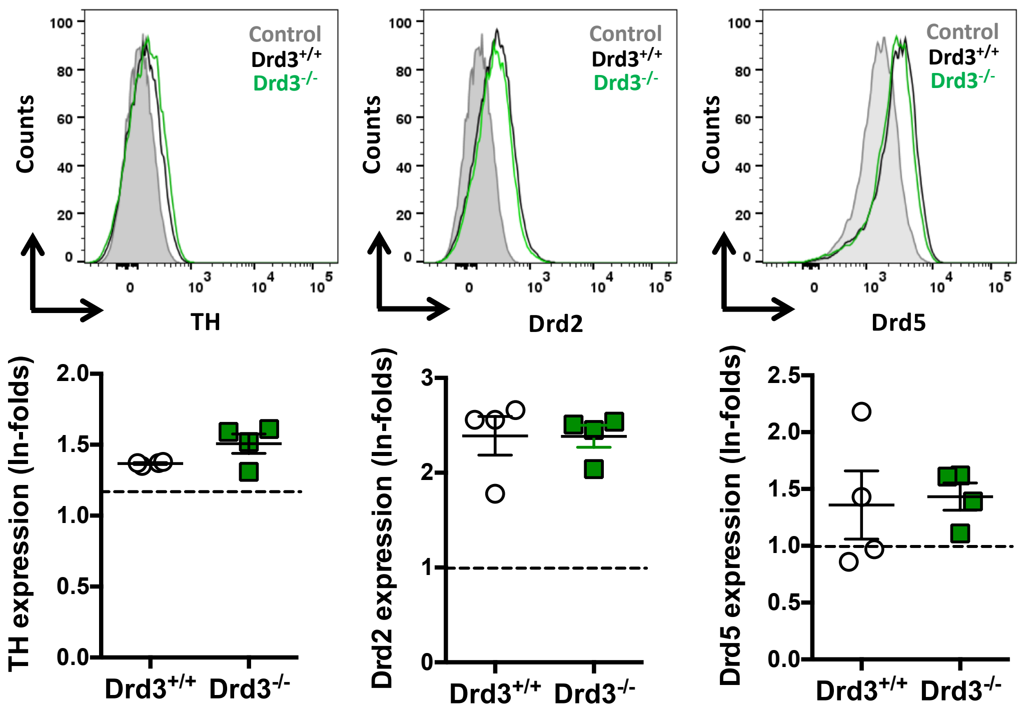

Figure S5. *Drd3* deficiency does not affect the expression of other components of the dopaminergic system in B-cells (associated to figure 1). The expression of tyrosine hydroxylase (TH, left panels), dopamine receptor D2 (Drd2, middle panels) and dopamine receptor D5 (Drd5, right panels) was analyzed in the $CD19^+$ population in *Drd3*-sufficient (black lines) and *Drd3*-deficient (green lines) splenic B-cells by flow cytometry. TH immunostaining was performed in permeabilized cells whilst Drd2 and Drd5 immunostaining was carried out in non-permeabilized cells. Top panels show representative histograms. The grey histograms represent negative controls of immunostaining: an isotype-matched control in the case of TH (left panel), or the pre-treatment of the anti-Drd2 or anti-Drd5 antibodies with the specific antigenic peptide recognized by the respective antibody (middle and right panels). Bottom panels show the quantification of the MFI associated to the immunostaining normalized by the MFI associated to negative control (In-fold). The dotted line shows in-fold = 1, which indicates no expression. Each symbol represents data obtained from an individual mouse; n = 4 mice per group. The mean ± SEM are depicted. Not significant differences were detected between both genotypes.

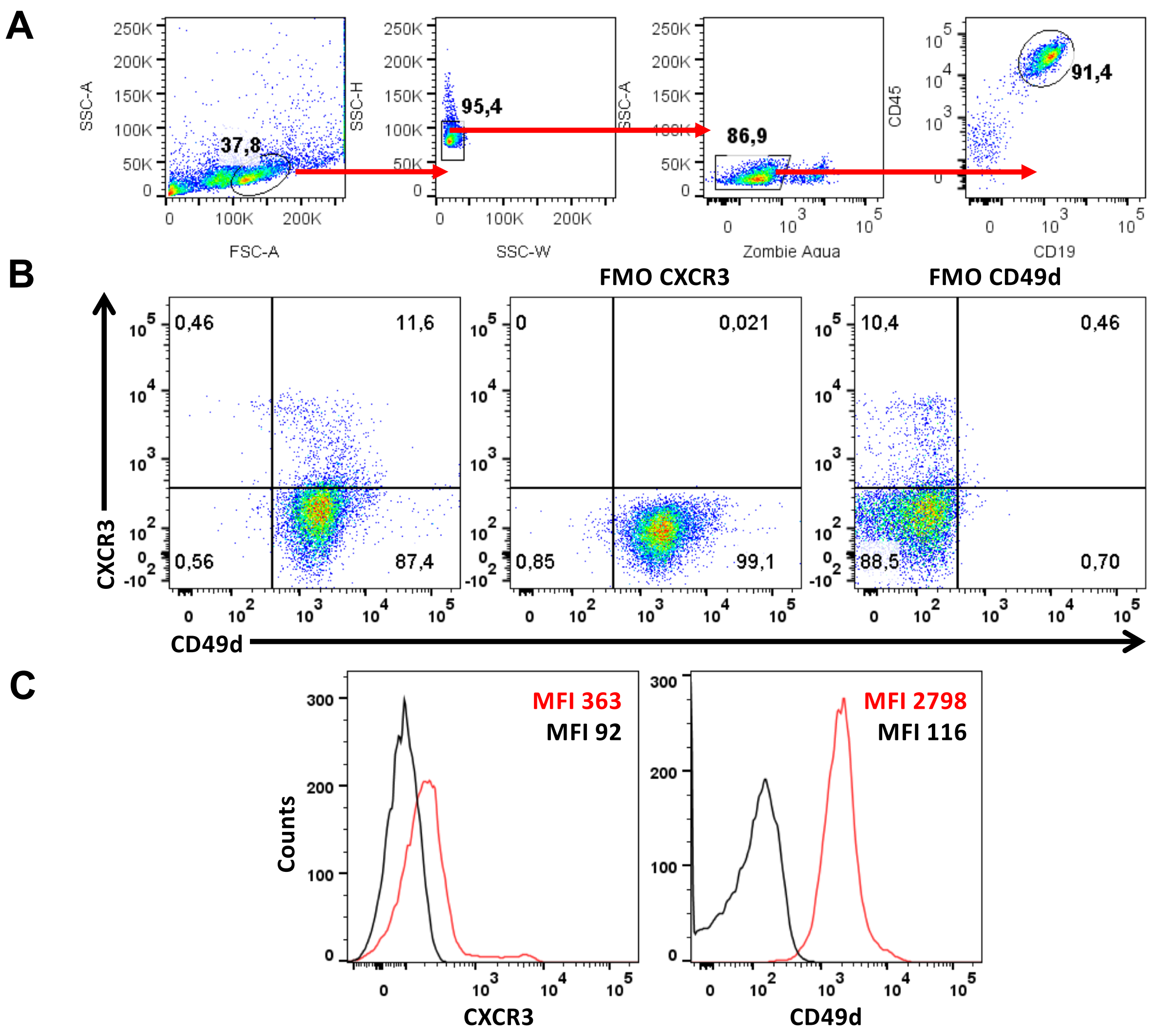

Figure S6. Gating strategy to analyse surface expression of homing molecules in B-cells (associated to figure 4 and 5). (**A**) Representative dot-plots showing the gating strategy to analyse surface expression of homing molecules in splenic B-cells isolated from Drd3 sufficient mice. Numbers indicate the percentage of cells inside the selected region. (**B**) Representative dot plots showing the analysis of expression of homing molecules in B-cells selected as in (A). Left panel shows the fluorescence associated to CXCR3 (BV421) and CD49d (PE) immunostaining. Middle panel shows the FMO for the BV421 channel, whilst right panel shows the FMO for the PE channel. Numbers indicate the percentage of cells inside the corresponding quadrant. (**C**) Representative histograms showing the fluorescence associated to CXCR3 immunostaining (left panel) and CD49d immunostaining (right panel). Black lined histograms represent fluorescence associated to FMO, whilst red lined histograms represent fluorescence associated to CXCR3 and CD49d immunostaining respectively.

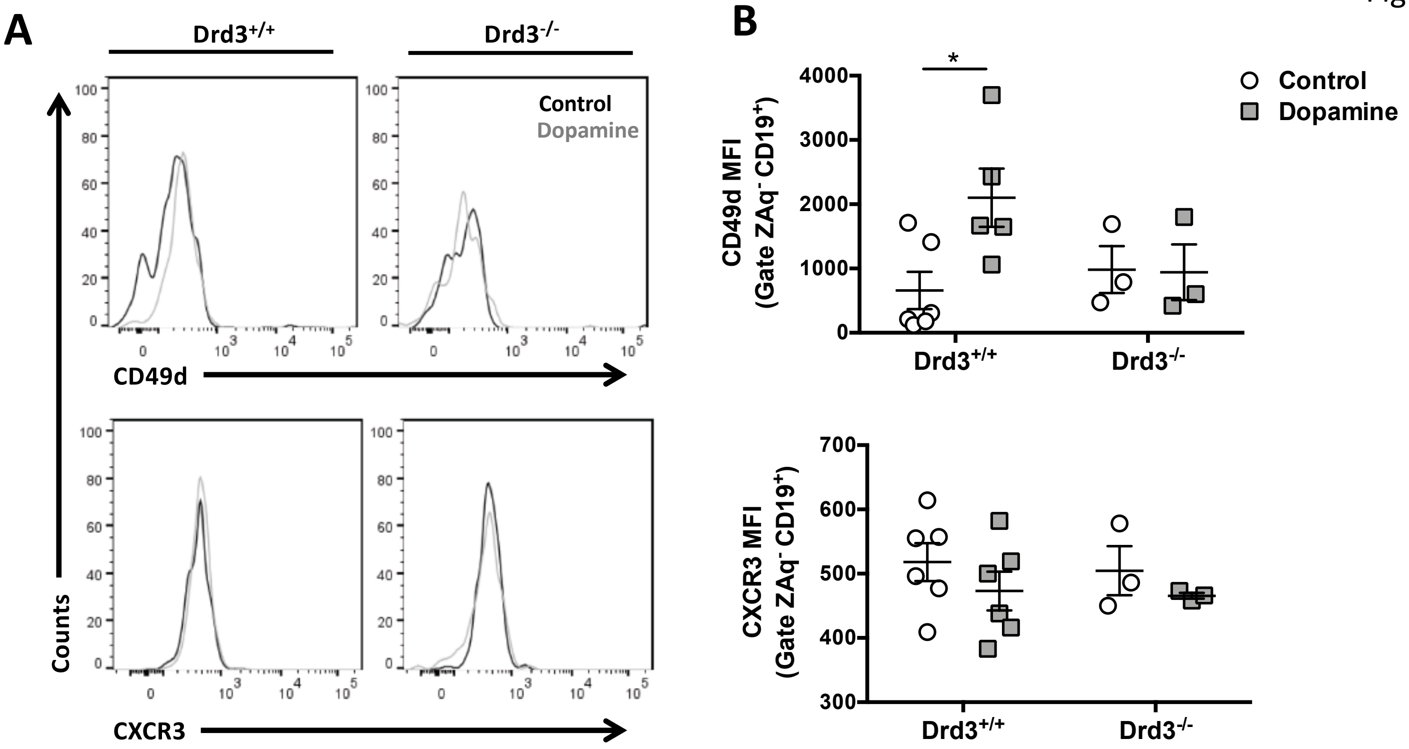

Figure S7. Drd3-signalling increases CD49d expression in B-cells (associated to figure 4). $CD19^{+}$ IgM^{int} $CD11c^{-}$ $TCR\beta^{-}$ cells were isolated by cell-sorting and then *in vitro* activated in the absence (control) or in the presence of 500 nM Dopamine (Dopamine) for 48h. Afterwards, the extent of surface expression of CXCR3 (left panel) or CD49d (right panel) was analysed by flow cytometry. (**A**) Representative histograms. (**B**) Quantification. Values are the mean fluorescence intensity (MFI) associated to the immunostaining of CXCR3 (left panel) or CD49d (right panel) of ZAq^{-} $CD19^{+}$ cells. Each symbol represents data obtained from an individual mouse; n = 3-6 mice per group. The mean ± SEM are depicted. *, $p<0.05$ by two-way ANOVA followed by Sidak's post-hoc test.

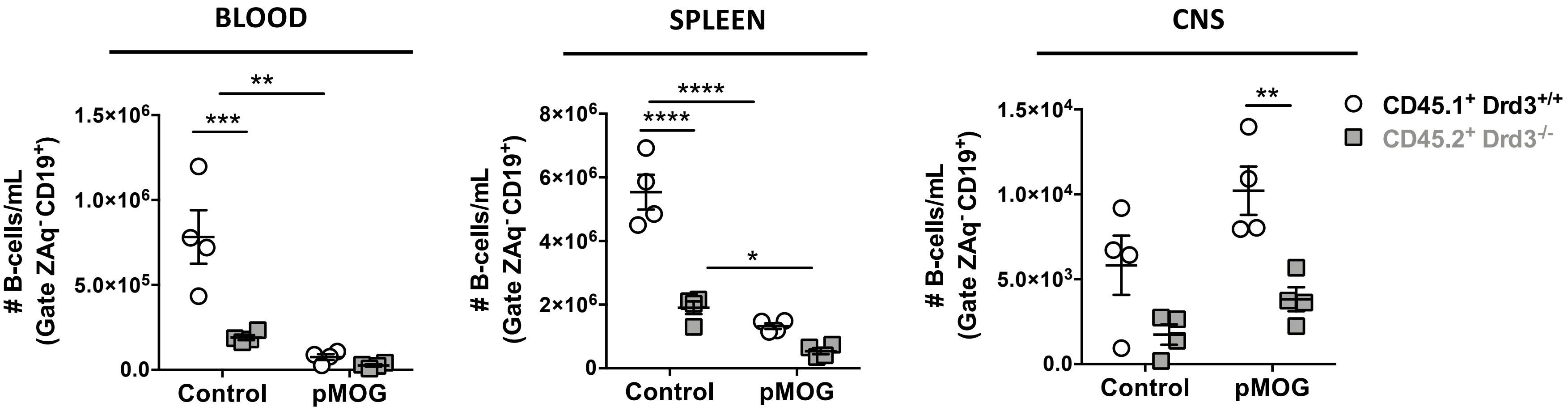

Figure S8. *Drd3* deficiency results in reduced number of B-cells under homeostasis or upon EAE induction (associated to figure 4). Chimeric mice were generated and treated as shown in figure 4D and, at the peak of disease severity (day 15 post-induction), mononuclear cells were isolated from peripheral blood (left panel), the spleen (middle panel) and the CNS (right panel) and the absolute number of $CD19^+$ B-cells was quantified by flow cytometry. Each symbol represents data obtained from an individual mouse; n = 4 mice per group. The mean ± SEM are depicted. *, p<0.05; **. P<0.01; ***, p<0.001, ****, p<0.0001 by two-way ANOVA followed by Sidak's *post-hoc* test.

Figure S9. Prado et al.

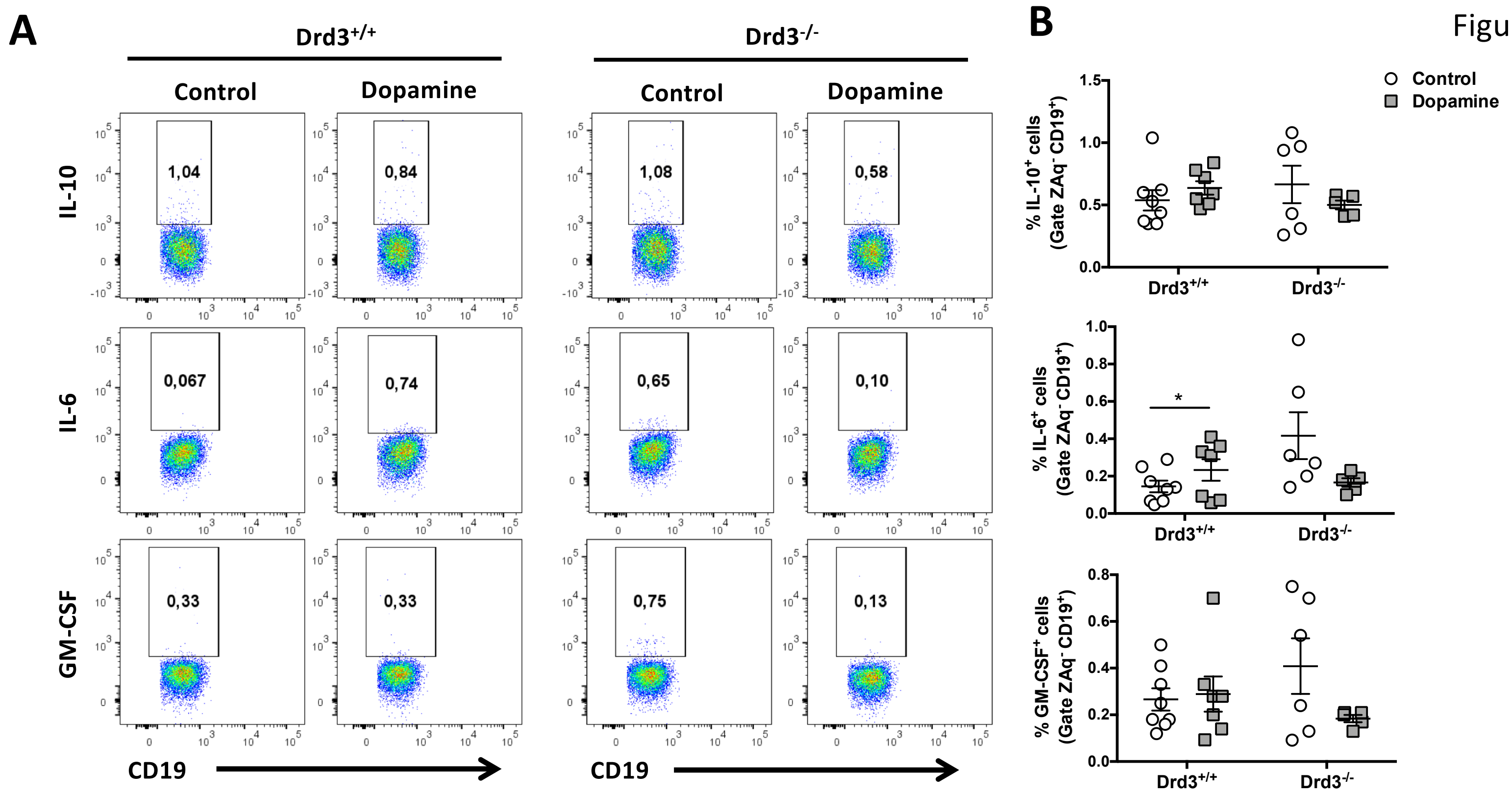

Figure S9. Analysis of cytokine production by activated B-cells in the presence of dopamine (associated to figure 4). *Drd3*-sufficient or *Drd3*-deficient $CD19^+$ IgM^{int} $CD11c^-$ $TCR\beta^-$ cells were isolated from the spleen by cell-sorting and then *in vitro* activated in the absence (control) or in the presence of 500 nM Dopamine (Dopamine) for 5 d. Afterwards, cells were re-stimulated with PMA and ionomycin in the presence of brefeldin A for 4 h and the extent of IL-10, IL-6 and GM-CSF production was assessed by intracellular cytokine immunostaining and analysed by flow cytometry. (**A**) Representative dot-plots are shown. (**B**) Quantification. Values are the percentage of cells producing the corresponding cytokine in the ZAq^- $CD19^+$ gate. Each symbol represents data obtained from an individual mouse; n = 5-7 mice per group. The mean ± SEM are depicted. *, $p<0.05$ by two-way ANOVA followed by Sidak's post-hoc test.

Figure S10. Prado et al.

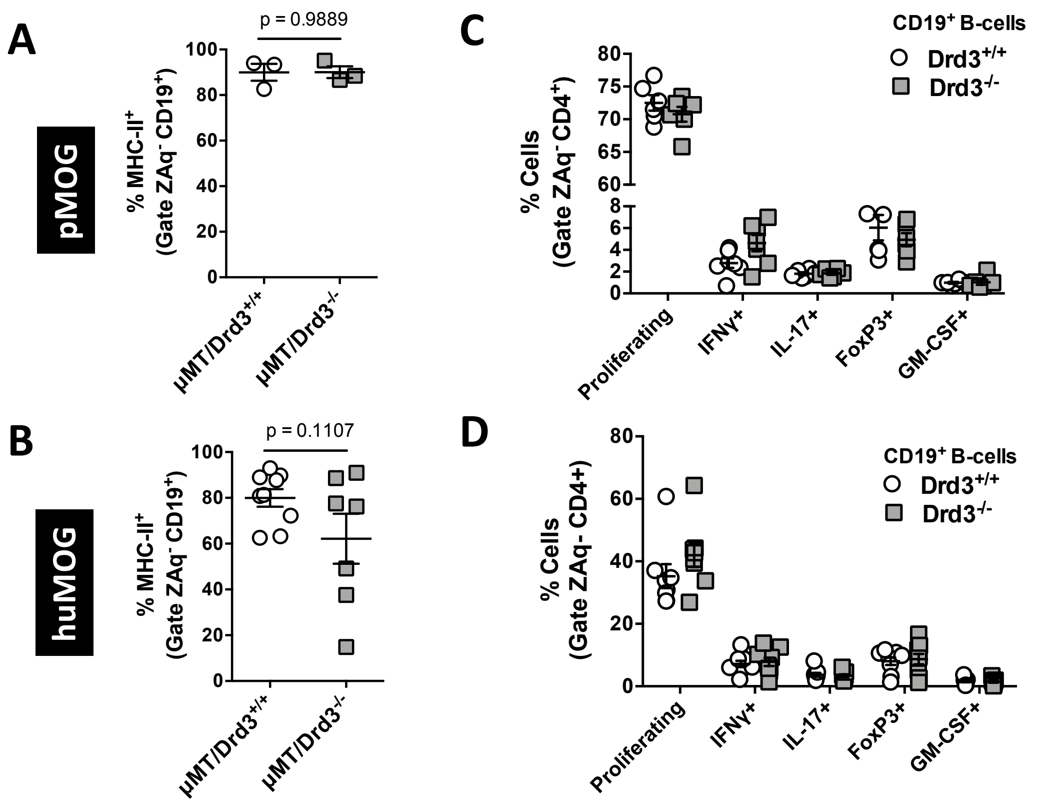

Figure S10. *Drd3*-deficiency in B cells does not impair antigen-presentation to T-cells (associated to figures 4 and 5). (A and B) MHC-II expression was evaluated by Flow cytometry analysis in splenic $CD19^+$ B-cells obtained from μMT/$Drd3^{+/+}$ or μMT/$Drd3^{-/-}$ chimeric mice immunized with (**A**) pMOG or (**B**) huMOG. (C-D) *In vitro* antigen-presentation assays. 2D2 $CD4^+$ T-cells, which express the transgenic TCR specific for recognizing the peptide $pMOG_{35\text{-}55}$ on IA^b, were loaded with the fluorescent probe Cell Trace Violet (CTV). B-cells were pulsed with (**C**) pMOG-beads or (**D**) huMOG-beads overnight and then co-cultured with CTV-loaded 2D2 $CD4^+$ T-cells. After 5 days, proliferation as well as cytokine production were determined by flow cytometry in living (ZAq^-) $CD4^+$ T-cells. Each symbol represents data obtained from an individual mouse; n = 3-9 mice per group. The mean ± SEM are depicted. Not significant differences were detected between both genotypes.

Figure S11. Prado et al.

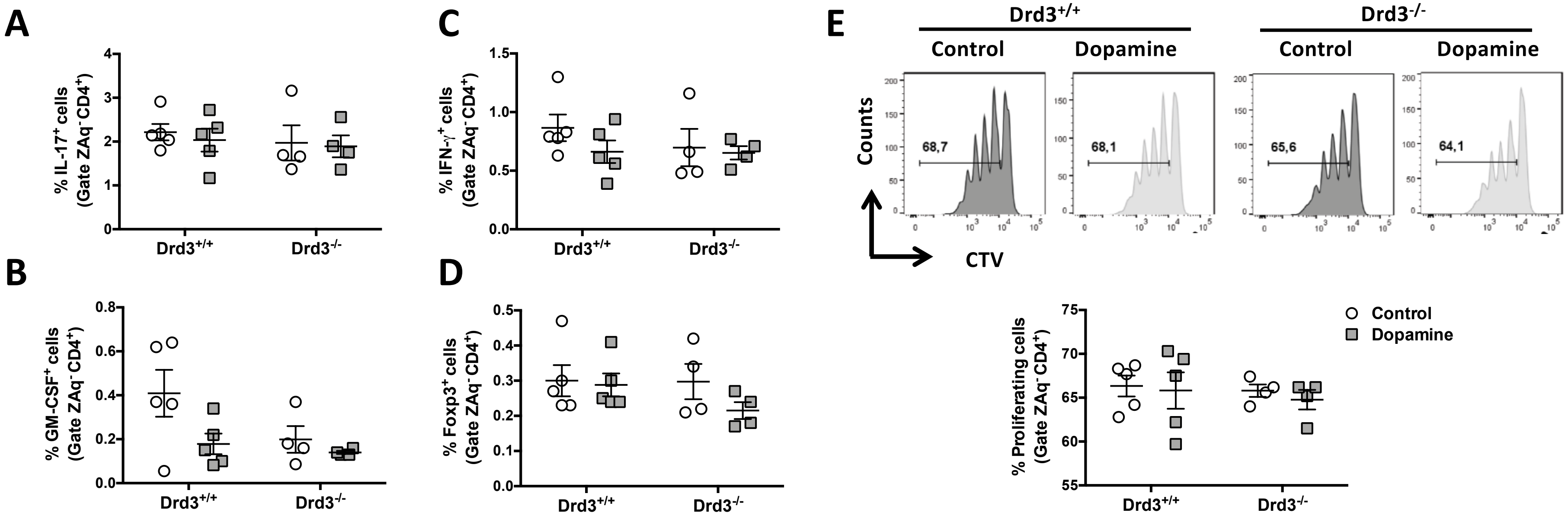

Figure S11. (associated to figure 5). Drd3-signalling in B-cells acting as APC does not affect the extent of cytokine production by T-cells. *Drd3*-sufficient or *Drd3*-deficient $CD19^+$ IgM^{int} $CD11c^-$ $TCR\beta^-$ cells were isolated by cell-sorting and then *in vitro* pulsed with the antigen in the absence (control) or in the presence of 500 nM Dopamine (Dopamine) for 2h. Afterwards, B-cells were washed and co-cultured with 2D2 $CD4^+$ T-cells, which were previously loaded with the fluorescent probe Cell Trace Violet (CTV). After 5 days, cells were re-stimulated with PMA and ionomycin for 5 h in the presence of brefeldin A, and the extent of IL-17 (**A**), GM-CSF (**B**) and IFN-***γ*** (**C**) production was determined by intracellular cytokine staining in the living (ZAq^-) CD4+ population. The expression of Foxp3 in the living (ZAq^-) $CD4^+$ T-cell population was assessed by intracellular immunostaining followed by flow cytometry (**D**). The extent of proliferation of living (ZAq^-) CD4+ T-cells was evaluated as the percentage of cells displaying dilution of the fluorescence associated to CTV by flow cytometry (**E**). Top panel shows representative histograms; bottom panel shows the quantification. (A-E) Each symbol represents data obtained from an individual mouse; n = 4-5 mice per group. The mean ± SEM are depicted. Not significant differences were detected between genotypes or treatments.
